# Egfl6 promotes ovarian cancer progression by enhancing the immunosuppressive functions of tumor-associated myeloid cells

**DOI:** 10.1172/JCI175147

**Published:** 2024-11-01

**Authors:** Sarah Hamze Sinno, Joshua A. Imperatore, Shoumei Bai, Noémie Gomes-Jourdan, Nyasha Mafarachisi, Claudia Coronnello, Linan Zhang, Eldin Jašarević, Hatice U. Osmanbeyoglu, Ronald J. Buckanovich, Sandra Cascio

**Affiliations:** 1 Magee-Womens Research Institute, Pittsburgh, Pennsylvania, USA.; 2 Division of Gynecologic Oncology, Department of Obstetrics, Gynecology, and Reproductive Sciences, University of Pittsburgh, Pittsburgh, Pennsylvania, USA.; 3 Carnegie Mellon University, Pittsburgh, Pennsylvania, USA.; 4 Ri.MED Foundation, Palermo, Italy.; 5 Department of Applied Mathematics, School of Mathematics and Statistics, Ningbo University, Ningbo, Zhejiang, China.; 6 Department of Computational and Systems Biology, Pittsburgh, Pennsylvania, USA.; 7 Department of Biomedical Informatics, School of Medicine,; 8 UPMC Hillman Cancer Center,; 9 Department of Bioengineering, School of Engineering, and; 10 Division of Hematology/Oncology, Department of Medicine, University of Pittsburgh School of Medicine, Pittsburgh, Pennsylvania, USA.

**Keywords:** Immunology, Oncology, Cancer immunotherapy, Macrophages, Obstetrics/gynecology

## Abstract

Tumor-associated macrophages (TAMs) and myeloid-derived suppressor cells (MDSCs) play a critical role in resistance to immunotherapy. In this study, we identified epidermal growth factor-like 6 (Egfl6) as a regulator of myeloid cell functions. Our analyses indicated that Egfl6, via binding with β3 integrins and activation of p38 and SYK signaling, acts as a chemotactic factor for myeloid cell migration and promotes their differentiation toward an immunosuppressive state. In syngeneic mouse models of ovarian cancer (OvCa), tumor expression of Egfl6 increased the intratumoral accumulation of polymorphonuclear (PMN) MDSCs and TAMs and their expression of immunosuppressive factors, including *CXCL2*, *IL-10,* and *PD-L1*. Consistent with this, in an immune ‘hot’ tumor model, Egfl6 expression eliminated response to anti-PD-L1 therapy, while Egfl6 neutralizing antibody decreased the accumulation of tumor-infiltrating CD206^+^ TAMs and PMN-MDSCs and restored the efficacy of anti-PD-L1 therapy. Supporting a role in human tumors, in human OvCa tissue samples, areas of high EGFL6 expression colocalized with myeloid cell infiltration. scRNA-Seq analyses revealed a correlation between *EGFL6* and immune cell expression of immunosuppressive factors. Our data provide mechanistic insights into the oncoimmunologic functions of EGFL6 in mediating tumor immune suppression and identified EGFL6 as a potential therapeutic target to enhance immunotherapy in patients with OvCa.

## Abstract 2

Tumor expression of Egfl6 induces the accumulation of intra-tumoral PMN-MDSC and TAM and enhance their immune suppressive activities inhibiting the response to anti-PD-L1.

## Graphical abstract

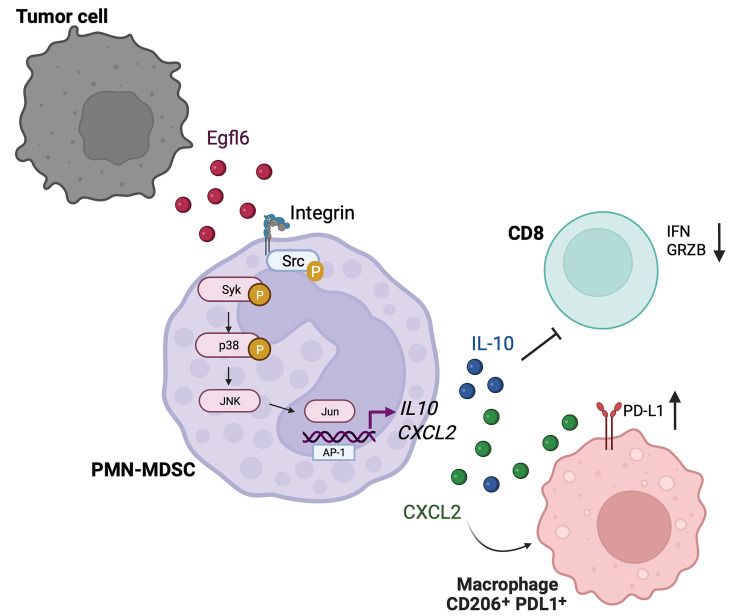

## Introduction

The introduction of immune checkpoint inhibitor (ICI) therapy to the clinic has transformed patient cancer care. The considerable response and survival benefit in certain types of cancer, including melanoma and lung cancer, have led to an increasing number of studies focused on the characterization of signaling pathways and identification of factors that drive resistance to current ICI therapies, with the goal of developing more efficient immunotherapeutic approaches.

Despite the benefits of ICI therapy, even in highly responsive tumors such as melanoma, most patients do not respond to ICI therapy (1, 2). Additionally, in many tumors such as ovarian cancer (OvCa), only 10%–20% of patients respond to ICI therapy. Thus, more effective immunotherapies are needed (3, 4). One reason for the limited efficacy of immune therapies in many cancers, including OvCa, may be the immunosuppressive tumor microenvironment (TME), which is characterized by a large number of tumor-associated myeloid cells, including myeloid-derived suppressor cells (MDSCs) and tumor-associated macrophages (TAMs) (5–11). MDSCs, which are known to promote angiogenesis, tumor progression, and metastasis, are divided into 2 subgroups, granulocytic/polymorphonuclear MDSCs (PMN-MDSCs) and monocytic MDSCs (M-MDSCs) (12).

The number of PMN-MDSCs and M-MDSCs in the peripheral blood of patients with cancer, including patients with OvCa, positively correlates with cancer stage and metastasis (8, 13–15). Moreover, high numbers of ascites- and tumor-infiltrating MDSCs are associated with poor prognosis in patients with high-grade serous OvCa (HGSOC) (16, 17). Both tumor-infiltrating PMN-MDSCs and M-MDSCs exhibit strong immune-suppressive activity toward T cells and natural killer (NK) cells by inhibiting both their proliferation and their effector functions. Some mediators of their immune suppressive activities are Arginase I (Arg), nitric oxide, and reactive oxygen species (18–20). Additionally, intratumoral MDSCs regulate antitumor immune response by promoting an M2-like phenotype of TAMs via secretion of IL-1 and IL-10 (21). Moreover, circulating M-MDSCs that migrate into the TME can differentiate into TAMs, which maintain major characteristics of their precursors, including a persistent expression of S100A8/A9 and immune-suppressive activity (22, 23). Like MDSCs, TAMs can induce immune suppression and are associated with poor prognosis in most solid tumors, including OvCa (9, 24). Indeed, in murine studies, depletion of MDSCs or TAMs allows activation of antitumor immune response, reducing tumor growth and progression (8). Taken together, these studies suggest regulators of MDSCs/TAMs could be important therapeutic targets.

Human epidermal growth factor-like 6 (EGFL6) protein is a candidate regulator of immune cell migration and differentiation. In cancer and in development, EGFL6 regulates differentiation of many cell types in a paracrine and autocrine manner, including osteoblasts (25), cancer stem-like cells (26), and adipocytes (27). EGFL6 is expressed in tumor endothelial cells as well as in cancer epithelial cells of the breast, colon, and ovarian tumors (26, 28, 29). EGFL6 expression is substantially elevated in HGSOC and is associated with poor patient prognosis (26, 30, 31). EGFL6 also promotes both endothelial cell migration during angiogenesis and cancer cell migration to drive cancer metastasis (28, 29).

Structurally, EGFL6 presents 3 intact and 1 partial EGF-like repeats, and a Arg-Gly-Asp (RGD) integrin-binding motif (32). It has been reported that the RGD motif mediates EGFL6 cellular signaling in both epithelial and endothelial cells by interacting directly with β1 and β3 integrins (26, 32, 33). Upon binding with integrins, EGFL6 promotes activation of several intracellular pathways, including pSHP2/p-ERK (26), PIK3/AKT (33) or BMP-Smad and mitogen-activated protein kinase (MAPK) signaling (25).

Here, we evaluate, for the first time, the impact of EGFL6 on tumor innate and adaptive immune response. We find that human and murine Egfl6, via activation of β integrins, induce Syk phosphorylation and promote myeloid cell differentiation toward an immunosuppressive state. Using syngeneic 2F8c, ID8, and ID8*^p53–/– Brca2–/–^* OvCa mouse models and human OvCa tissue samples, we found that tumor Egfl6 induces the accumulation of intra-tumoral MDSCs and TAMs. Notably, in the immune-responsive 2F8c mouse model, tumor Egfl6 expression induces resistance to anti-PD-L1 (a-PD-L1) therapy. In contrast, Egfl6 neutralizing antibody (NAb) therapy enhanced the efficacy of ICI both in the 2F8c and ID8*^p53–/– Brca2–/–^* models. We propose Egfl6 as a potential target to overcome the immunoinhibitory effects of the TME and improve the efficacy of ICI in the treatment of HGSOC.

## Results

### Egfl6 promotes the differentiation of granulocytic myeloid cells.

**Figure 1:**
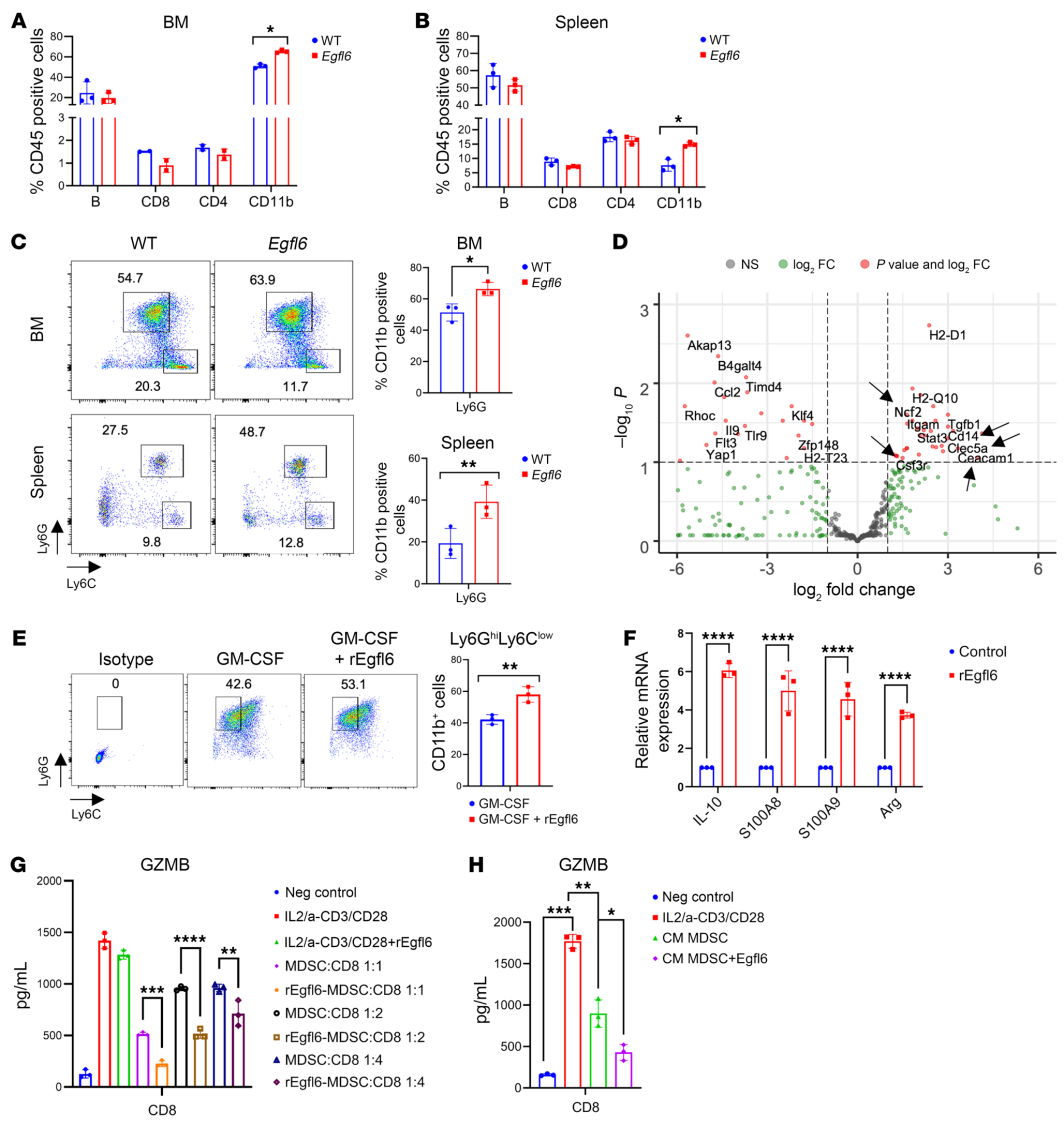
*Egfl6* mice display an increased number of BM and splenic myeloid cells. (**A** and **B**) Graphs represent the percentage of B, CD4^+^, CD8^+^, and CD11b^+^ cells in BM (**A**) and spleen (**B**) of WT and *Egfl6* mice. (**C**) Gating and quantification of Ly6G and Ly6C subsets of CD11b^+^ BM and splenic cells from healthy C57BL/6J (WT) and *Egfl6* mice. (**D**) Volcano plot showing differentially expressed genes (DEGs) between BM CD11b^+^ cells of *Egfl6* mice versus C57BL/6J (WT). *P* values determined via *t* test are plotted on the *y* axis. DEGs are colored in red. (**E**) Gating and quantification of BM-derived CD11b^+^Ly6G^+^Ly6C^–^ cells stimulated with rGM-CSF ± rEgfl6. (**F**) qPCR analyses of indicated genes in sorted BM CD11b^+^ cells stimulated with rGM-CSF + rEgfl6. Stimulation with rGM-CSF alone was used as control. (**G** and **H**) ELISA of Granzyme B (GZMB) in IL-2 + CD3/CD28 activated CD8^+^ T cells and cultured directly with rEgfl6-stimulated BM-derived MDSC cells or MDSC control at different ratio (**G**) or with the conditioned media (CM) of rEgfl6-stimulated BM-derived MDSC cells or MDSC control (**H**). Unstimulated CD8^+^ T cells were used as negative control. Results were analyzed using unpaired 2-tailed *t* test or 2-way ANOVA. Experiments were performed in triplicate. Data are presented as mean ± SEM. *P < 0.05, **P < 0.01, ***P < 0.001, ****P < 0.0001.

To investigate the impact of Egfl6 on the immune system, we first used mice with CRE-inducible *Egfl6* expression at the Rosa26 locus Rosa^LSL–Egfl6^ crossed with CMV-CRE mice. The resulting Rosa26promoter-driven *Egfl6* mice, here referred to as *Egfl6* mice, broadly express *Egfl6*. Flow cytometry analysis revealed that *Egfl6* mice, compared with the control C57BL/6J (WT) mice, have a higher number of CD11b^+^ cells both in the bone marrow (BM) (Figure 1A) and in the spleen (Figure 1B). No significant difference was detected in the number of B, CD8^+^, and CD4^+^ T cells, though there was a trend for decreased B and CD8^+^ T cells (Figure 1, A and B). Representative gating strategy and flow cytometry analysis of BM from WT and *Egfl6* mice are shown in Supplemental Figure 1, A and B; supplemental material available online with this article; https://doi.org/10.1172/JCI175147DS1 More detailed flow cytometry analyses revealed an increased number of granulocytic cells (CD11b^+^ Ly6G^Hi^Ly6C^Lo^) in both the BM and spleen of *Egfl6* mice compared with control WT mice (Figure 1C). To examine the transcriptional landscape of BM myeloid cells in *Egfl6* mice, we measured the expression of 754 genes involved in the innate immune response using the NanoString nCounter Mouse Myeloid Innate Immune Panel on CD11b^+^ cells magnetically sorted from the BM of *Egfl6* and WT mice. We observed that: (a) genes associated with both the differentiation and function of granulocytes, such as colony stimulator factor 3 receptor (*Csfr3*), neutrophil cytosolic factor 2 (*Ncf2*), C-type lectin domain containing 5A (*Clec5a*), and carcinoembryonic antigen-related cell adhesion molecule 1 (*Ceacam1*), and (b) genes associated with monocytes, including *CD14*, showed increased expression in the BM myeloid cells of *Egfl6* mice compared with the control mice (Figure 1D).

Given that broad Egfl6 expression in the mouse model is nonphysiologic, we then confirmed the results ex vivo, using BM treatment with Egfl6 fusion protein. To determine whether Egfl6 could directly modulate the differentiation of myeloid cells, BM CD11b^+^ cells were isolated from healthy WT mice and cultured for 5 days in the presence of granulocyte-macrophage colony-stimulating factor (GM-CSF) and/or murine recombinant Egfl6 protein (rEgfl6). Stimulation with rEgfl6 increased the number of CD11b^+^Ly6G^Hi^Ly6C^Lo^ cells (Figure 1E). Consistent with the RNA-Seq results, qRT-PCR on BM Gr-1^+^ cells isolated from WT mice and stimulated with rEgfl6 indicated that Egfl6 promotes the gene expression of *Clec5a* and *Csfr3* in granulocytes (Supplemental Figure 1C).

Next, we investigated whether Egfl6 could modulate myeloid cell phenotype and functional activities. BM myeloid cells were differentiated into MDSCs in the presence of rEgfl6 as described in Methods. Stimulation of MDSC with rEgfl6 significantly increased the gene expression of the immunosuppressive factors *IL-10,* *S100A8/9*, and *Arginase* (*Arg*) (Figure 1F). To determine whether Egfl6 regulates the immunosuppressive activity of myeloid cells, we performed an ELISA assay to evaluate whether Egfl6 treatment of MDSC could also modulate CD8^+^ T cells secretion of Granzyme B (GZMB), a factor associated with cytotoxic activity. Activated splenic CD8^+^ T cells were cocultured with Egfl6-stimulated BM-derived MDSC or control MDSCs at different ratios. Egfl6-treated MDSCs reduced the secretion of GZMB in CD8^+^ T cells compared with MDSC controls (Figure 1G). In addition, we performed a CD8^+^ T cell proliferation assay. The proliferation of CD8^+^ T cells was lower in the presence of Egfl6-treated MDSCs compared with MDSCs alone (Supplemental Figure 1D).

To determine if the impact of Egfl6 was on MDSC secreted factors, we repeated this experiment but treated T cells with conditioned medium (CM) of Egfl6-stimulated BM-derived MDSCs or controls. CM of Egfl6-stimulated BM-derived MDSCs displayed lower secretion of GZMB and Perforin compared with CD8^+^ T cells cultured with CM of MDSC controls (Figure 1H and Supplemental Figure 1E). Notably, treatment of CD8^+^ T cells with rEgfl6 did not affect GZMB secretion or proliferation (Figure 1G and Supplemental Figure 1D) suggesting that Egfl6 induces immunosuppression indirectly by modulation of MDSC activities.

### Egfl6-expressing tumors display accelerated tumor growth and an increased number of intratumoral immunosuppressive MDSCs and TAMs.

To evaluate the impact of tumor cell–expressed Egfl6 on the tumor immune microenvironment, we stably expressed Egfl6 in the 2F8c (2F8c-Egfl6) and ID8 (ID8-Egfl6) murine OvCa cell lines, both of which can be grown syngeneically in C57BL/6J (WT) mice. Moreover, the 2F8c OvCa cell line generates ICI-responsive tumors abundantly infiltrated by CD3^+^ lymphocytes (34, 35) whereas ID8-derived tumors display a cold/immune desert profile (36, 37) unresponsive to single ICI treatment (38–40). The expression of Egfl6 was confirmed via qPCR and ELISA (Supplemental Figure 2A).

**Figure 2:**
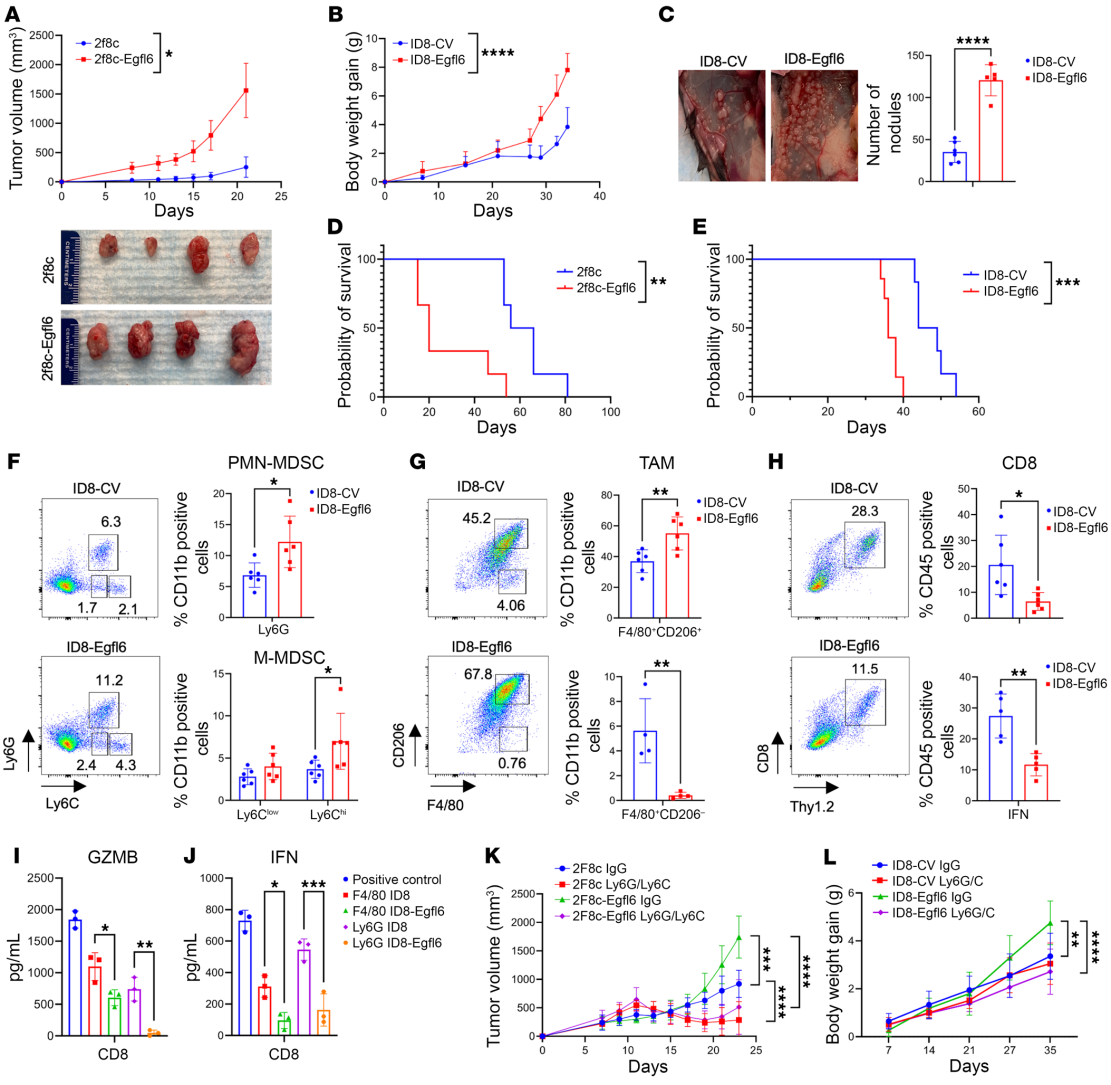
Egfl6 accelerates tumor growth and modulates the immune TME. (**A**) Tumor volume changes (mm^3^) and images of 2F8c and 2F8c-Egfl6 subcutaneous tumors resected and measured 3 weeks after tumor cell inoculation (*n* = 6 mice per group). (**B**) Time-dependent body weight gain in mice i.p. injected with ID8-CV and ID8-Egfl6 tumors (*n* = 8 mice per group). (**C**) Evaluation of peritoneal metastases of ID8-CV and ID8-Egfl6 that had a weight increase of over 35% of their original weight on the day of tumor cell injections (*n* = 6 mice per group). (**D** and **E**) Kaplan-Meier overall survival analysis for 2F8c+/–Egfl6 (**D**) and ID8+/–Egfl6 (**E**). Survival statistics were calculated using log-rank analysis from Kaplan-Meier survival plots. (**F** and **G**) Flow cytometric evaluation and summary of PMN-MDSC (CD11b^+^Ly6G^+^Ly6C^–^) (**F**, top panel), M-MDSC (CD11b^+^Ly6G^–^Ly6C^+^) (**F**, bottom panel), and TAM (CD11b^+^F4/80^+^CD206^+^) (**G**) in ID8+/–Egfl6 tumors. (**H**) Flow cytometric evaluation and quantification of CD8 T (CD45^+^Thy1.2^+^) cells and their expression of IFN-γ in ID8+/–Egfl6 tumors. (**I** and **J**) ELISA of Granzyme B (GZMB) (**I**) and IFN (**J**) in IL-2 + CD3/CD28 activated CD8 T cells (Pos Control) and cultured directly with F4/80^+^ or Ly6G^+^ cells isolated from ID8 and ID8-Egfl6 ascites at ratio of 1:1. (**K** and **L**) Time-dependent volume changes (mm^3^) of 2F8c and 2F8c-Egfl6 tumor cells (**K**) or body-weight gain in mice i.p. injected with ID8 and ID8-Egfl6 tumor cells (**L**) and treated with anti-Ly6G/Ly6C Ab or IgG isotype control (*n* = 6 mice per group). *P* values were calculated using unpaired 2-tailed *t* test, 1-way, or 2-way ANOVA with Tukey’s post test for multiple comparisons. Experiments were performed in triplicate. Data are presented as mean ± SEM. *P < 0.05, **P < 0.01, and ***P < 0.001, ****P < 0.0001.

We first evaluated the 2F8c-Egfl6 model, in which we found that expression of Egfl6 resulted in a significant increase in tumor growth (Figure 2A). We then repeated this using i.p. injection of the ID8 cells. Animal weight was used as a marker of ascites accumulation and disease progression. Compared with mice injected with control ID8 cells, ID8-Egfl6–injected mice gained body weight more rapidly (Figure 2B) and presented a higher number of metastatic nodules attached to the peritoneal wall (Figure 2C). Consistent with this, the overall survival of mice bearing 2F8c-Egfl6 (Figure 2D) or ID8-Egfl6 (Figure 2E) tumors was reduced compared with controls.

Next, we analyzed the abundance and phenotype of tumor-infiltrating immune cells in the ID8 tumor-associated ascites. Flow cytometry analysis revealed that ID8-Egfl6 ascites had higher accumulation of PMN-MDSCs (Figure 2F, top panel), defined as CD11b^+^Ly6G^+^Ly6C^Lo^, as well as M-MDSCs (Figure 2F, bottom panel), defined as CD11b^+^Ly6G^–^Ly6C^Hi^MHCII^Neg^. The number of immunosuppressive CD206^+^ M2-type TAMs was also higher in Egfl6^+^ tumors compared with tumor controls (Figure 2G, top panel). Interestingly, a CD206-negative macrophage population found in ID8-CV ascites was absent in ID8-Egfl6 ascites (Figure 2G, bottom panel). Consistent with an immunosuppressive TME, ID8-Egfl6^+^ tumor ascites had fewer CD8^+^ T cells than the control ascites (Figure 2H, top panel), and there was reduction in the percentage of CD8^+^ T cells producing IFN-γ (Figure 2H, bottom panel). Similar data were found in 2F8c-Egfl6 tumors, showing an increased numbers of intratumoral PMN-MDSCs and TAMs (Supplemental Figure 2, B and C) and decreased IFN-γ^+^ CD8^+^ T cells and CD107^+^ NK^+^ cells (Supplemental Figure 2D and E) compared with control tumors.

Egfl6 is known to promote tumor angiogenesis and is highly expressed in endothelial cells of human OvCa tissues (41). Consistent with a role in angiogenesis, tumor expression of Egfl6 increased the number of endothelial cells (Supplemental Figure 3A). Immunofluorescence assays indicated that Egfl6 was expressed in both tumor cells and weakly in endothelial cells (Supplemental Figure 3B).

### Myeloid cells play a key role in Egfl6-induced tumor progression.

Next, we evaluated the immunosuppressive function of PMN-MDSCs and TAMs isolated from ID8 and ID8-Egfl6 ascites. Ascites Ly6G^+^ and F4/80^+^ cells were sorted using magnetic beads and cultured with IL-2– and a-CD3/CD28–activated CD8^+^ T cells at 1:1 ratio. After 72 hours, we collected the supernatants and measured GZMB and IFN-γ protein secretion via ELISA. Ly6G^+^ and F4/80^+^ cells isolated from Egfl6^+^ ascites showed higher inhibition of GZMB and IFN-γ secretion compared with myeloid cells isolated from tumor controls (Figure 2, I and J). Altogether, these results indicate that myeloid cells isolated from Egfl6^+^ tumors have a higher immunosuppressive capacity compared with control tumors.

To evaluate the role of myeloid cells in Egfl6-driven tumor progression, 2F8c+/– Egfl6 or ID8+/– Egfl6 tumor-bearing mice were treated with IgG control or a-Ly6G/Ly6C antibody (Ab). a-Ly6G/Ly6C Ab treatment delayed tumor growth in both 2F8c+/– Egfl6 tumor-bearing mouse groups (Figure 2K). Importantly, when a-Ly6G/Ly6C Ab treatment was suspended, Egfl6^+^ 2F8c tumors started to grow again faster than 2F8c tumor control (Figure 2K). In the ID8 model, a-Ly6G/Ly6C Ab treatment significantly delayed Egfl6^+^ tumor growth (Figure 2L). These results further indicated that MDSCs are important for Egfl6-driven tumor growth. Upon Ly6G/Ly6C Ab treatment, the total number of CD11b^+^ cells was significantly reduced compared with IgG controls (Supplemental Figure 2F). While no PMN-MDSCs were detected in Ly6G/Ly6C-treated tumors, a low number of intratumoral monocytic cells were still infiltrating treated tumors (Supplemental Figure 2G). Notably, the number of F4/80^+^CD206^+^ macrophages was reduced upon the treatment (Supplemental Figure 2H) and they showed lower expression of PD-L1 (Supplemental Figure 2I). These data indicate that: (a) myeloid cells mediate, at least in part, the Egfl6-induced tumor progression; and (b) granulocytic/monocytic cells play a crucial role in the TAMs phenotype in Egfl6+ tumors.

### Egfl6 promotes migration of myeloid cells via β3 integrin.

EGFL6 has been previously shown to be involved in migration of endothelial cells and tumor cells (32, 42). To test whether EGFL6 could promote the migration of immune cells, we performed a transwell migration assay adding in the bottom chamber of the transwell plate either (a) complete RPMI media +/– rEGFL6 or (b) EGFL6-overexpressing SKOV3 human OvCa cells (SKOV3-EGFL6) or SKOV3 cells expressing the control vector (SKOV3-CV). PBMCs were resuspended in serum free media and plated in the top chamber. After 16 hours, migrated cells were analyzed by flow cytometry. Our data showed that rEGFL6 or SKOV3-secreted EGFL6 promoted the migration of CD14 cells by 35%–40% whereas the migratory activities of B cells, CD4^+^ or CD8^+^ T cells were not affected (Supplemental Figure 4A). Next, we assessed the impact of EGFL6 on the migration of myeloid cells isolated from ascites of patients with HGSOC. SKOV3-EGFL6 or SKOV3-CV were plated in the bottom chamber, and human CD33^+^ cells were plated in the top chamber. Migrated CD33^+^ cells were then identified via a-CD11b IHC. We observed that SKOV3-EGFL6 cells significantly enhanced the migratory activities of CD11b^+^CD33^+^ cells (Supplemental Figure 4B). Similarly, murine ID8 OvCa cells overexpressing Egfl6 (ID8-Egfl6) enhanced the migration of murine BM CD11b^+^ cells compared with ID8 cells stably transfected with a control vector (ID8-CV) (Supplemental Figure 4C). To confirm that Egfl6 directly enhances myeloid cells’ migratory activities, BM-isolated CD11b^+^ cells were stimulated with GM-CSF for 5 days and then plated in the top chamber. Addition of rEgfl6 on the bottom chamber enhanced myeloid cell migration by 50%–60% (Supplemental Figure 4D).

The integrin-binding RGD motif is known to be essential for the activity of Egfl6 in many settings (26, 32, 33). We previously reported that Egfl6 mediates tumor cell proliferation by binding integrin β3 (26). Thus, we determined whether β3 integrin could reduce the Egfl6-induced migratory activity in myeloid cells. Indeed, Cyclo-RGDfK (c-RGD), a potent α_v_β3 integrin inhibitor, reduced the migration of myeloid cells induced by ID8-Egfl6 cells and rEgfl6 (Supplemental Figure 4, C and D). This suggests that Egfl6 mediates migration of myeloid cells via β3 integrin.

### Egfl6 induces phosphorylation of Syk to promote activation of IL-10 and Cxcl2 in tumor-associated myeloid cells to drive immunosuppression.

**Figure 3:**
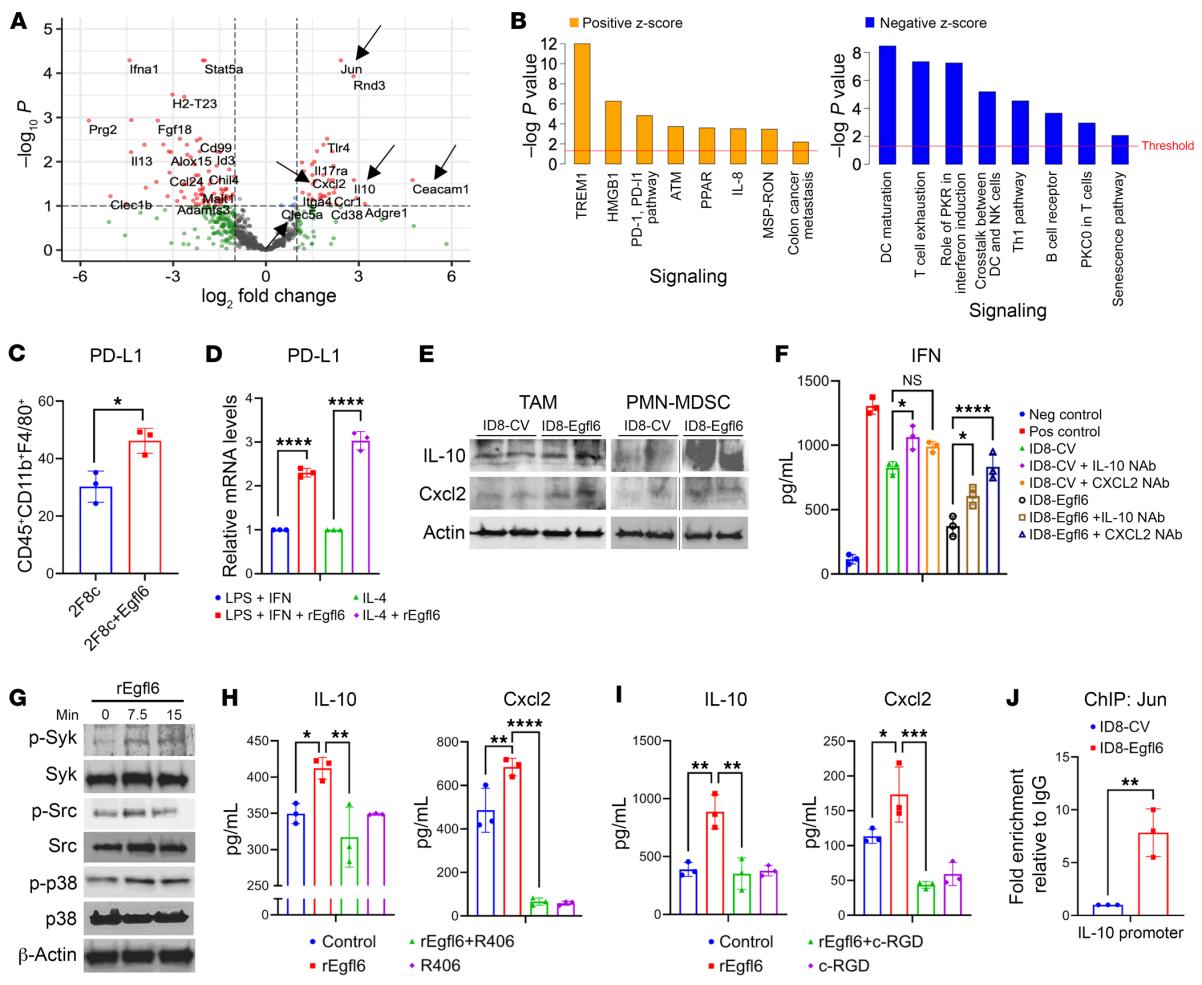
IL-10 and Cxcl2 mediate Egfl6 antitumor immunosuppression. (**A**) Volcano plot showing differentially expressed genes (DEGs) between CD11b^+^ cells infiltrating 2F8c-Egfl6 versus 2F8c tumors. Negative Log_10_ *P* values determined via *t* test are plotted on the *y* axis. (**B**) IPA protein analysis of Egfl6 treatment associated DEG pathways identified as significantly (*P* < 0.05) upregulated (left panel) or downregulated (right panel). (**C** and **D**) Summary of PD-L1 expression determined by flow cytometry in infiltrating TAMs (**C**) and by qPCR in BM-derived macrophages polarized with different stimuli as indicated **D**. (**E**) Western blotting analysis of IL-10 and Cxcl2 in TAMs and PMN-MDSCs isolated from ID8+/–Egfl6 ascites. Actin was used as loading control. (**F**) ELISA of IFN-γ in CD8^+^ T cells cultured with the Ly6G^+^ cells isolated from ID8+/–Egfl6 ascites in the absence/presence of IL-10 or Cxcl2 NAbs. (**G**) Western blotting showing the indicated protein expression in BM-isolated CD11b^+^ cells treated with GM-CSF and rEgfl6 for 0, 7.5, and 15 minutes. β-Actin was used as loading control. Results are representative of at least 3 independent experiments. (**H** and **I**) ELISA showing IL-10 and Cxcl2 protein secretion in GM-CSF-treated BM CD11b^+^ cells +/– rEgfl6 and/or Syk inhibitor (R406) (**H**), and GM-CSF-treated BM CD11b^+^ cells +/– rEgfl6 and/or the integrin inhibitor Cyclo-RGD (c-RGD) (**I**). (**J**) Graph represents a ChIP assay performed with anti-Jun Ab followed by qPCR to measure IL-10 promoter in ID8+/–Egfl6 ascites. Data are presented as mean ± SEM. *P* values were calculated using unpaired 2-tailed *t* test or 1-way ANOVA with Tukey’s post test for multiple comparisons. *P < 0.05, **P < 0.01, ***P < 0.001, and ****P < 0.0001. All results are representative of 3 independent experiments.

To gain insight into the function of myeloid cells recruited to Egfl6-expressing tumors, we evaluated the gene expression of 754 genes involved in the innate immune responses on CD11b^+^ cells isolated from 2F8c and 2F8c-Egfl6 tumors. Similar to the gene expression analysis of BM CD11b^+^ cells isolated from *Egfl6* mice, CD11b^+^ cells isolated from 2F8c-Egfl6 tumors displayed higher *IL10*, *Cxcl2*, *Clec5a*, and *Ceacam1* gene expression compared with tumor controls (Figure 3A). Ingenuity pathway analysis (IPA) of genes differentially expressed in cells from the 2F8c-Egfl6 tumors highlighted the (a) upregulation of signaling pathways linked with tumor progression and immunosuppression, including TREM1, HMGB1, IL-8(CXCL8)/CXCR2 axis (43), and PD-1/PD-L1 signaling (Figure 3B left panel) and (b) downregulation of Th1 immune response and DC maturation (Figure 3B, right panel). Flow cytometry analysis confirmed that, compared with tumor controls, PD-L1 was upregulated in TAMs of 2F8c-Egfl6 tumors (Figure 3C). To confirm that Egfl6 directly induces PD-L1 expression in macrophages, BM-derived macrophages were polarized into M1 and M2 in the presence of rEgfl6. rEgfl6 enhanced PD-L1 expression in both M1 (LPS^+^IFN-γ) and M2 (IL-4) by 2.3- and 3-fold, respectively (Figure 3D).

Both IL-10 and Cxcl2 are known to be implicated in the induction of immunosuppressive myeloid cell phenotype and inhibition of cytotoxic activities of CD8^+^ T cells (44–47). To identify the specific myeloid cell type secreting IL-10 and Cxcl2 in response to Egfl6, F4/80^+^ TAMs and Ly6G^+^ MDSCs were sorted from ID8+/–Egfl6 tumors and subjected to Western blotting. Our results indicate that, compared with tumor controls, TAMs infiltrating Egfl6^+^ tumors exhibited upregulation of Cxcl2 (Figure 3E, left panel), whereas PMN-MDSCs isolated from Egfl6^+^ tumors showed upregulation of both IL-10 and Cxcl2 (Figure 3E, right panel).

To test whether IL-10 and/or Cxcl2 inhibit the cytotoxic activity of CD8^+^ T cells in our model, activated splenic CD8^+^ T cells were cultured with Ly6G^+^ cells sorted from ascites of ID8-Egfl6 and ID8 tumor-bearing mice in the presence of IL-10 or Cxcl2 NAbs. ELISAs were performed to measure the amount of IFN-γ protein secretion. Our results indicated that Egfl6-induced CD11b^+^ cell immunosuppression was mitigated by IL-10 neutralization and completely overcome by Cxcl2 neutralization (Figure 3F).

To determine the molecular mechanism by which Egfl6 modulates gene expression in myeloid cells, we performed Western blotting using murine BM CD11b^+^ cells stimulated with GM-CSF+/– rEgfl6 or vehicle for 0, 7.5, and 15 minutes. After 7.5 minutes of stimulation, we observed rEgfl6 treatment increased phosphorylation of Syk, Src, and p38 (Figure 3G). As Syk is necessary for IL-10 production in dendritic cells (48) and neutrophils (49), we also evaluated the role of Syk activation in the Egfl6-driven induction of the IL-10 or Cxcl2 regulatory axis. The Syk-specific inhibitor R406 significantly inhibited both IL-10 and Cxcl2 protein expression (Figure 3H). Suggesting a role for β3 integrin binding in Egfl6-mediated effects, inhibition of β3 integrin drastically reduced Egfl6 induction of both IL-10 and Cxcl2 protein expression (Figure 3I).

Previous studies reported that *IL-10* gene expression is dependent on JNK protein and p38 activation (50), as well as AP1 transcriptional factors (51). An association between c-Jun, a component of AP1 family members, and the IL-10 promoter has been identified both in T cells and monocytes/macrophages (52). Indeed, an AP1 consensus site was identified at –1357 bp of the IL-10 promoter. Because Jun was highly expressed in CD11b^+^ cells of Egfl6^+^ tumors (Figure 3A), we evaluated whether Egfl6 induced the binding of Jun to the IL-10 promoter in myeloid cells. ChIP assay suggested that the binding of Jun on IL-10 promoter was significantly stronger in CD11b^+^ cells isolated from ID8-Egfl6 ascites compared with CD11b^+^ cells isolated from control ascites (Figure 3J). Thus, our data suggest that in myeloid cells, Egfl6, via β3 integrin, induces the activation of Src/Syk/p38 and enhances the association of Jun to the IL-10 promoter, which is necessary for its expression.

### Egfl6 expression by ovarian tumor cells reduces the efficacy of a-PD-L1 immune therapy.

**Figure 4:**
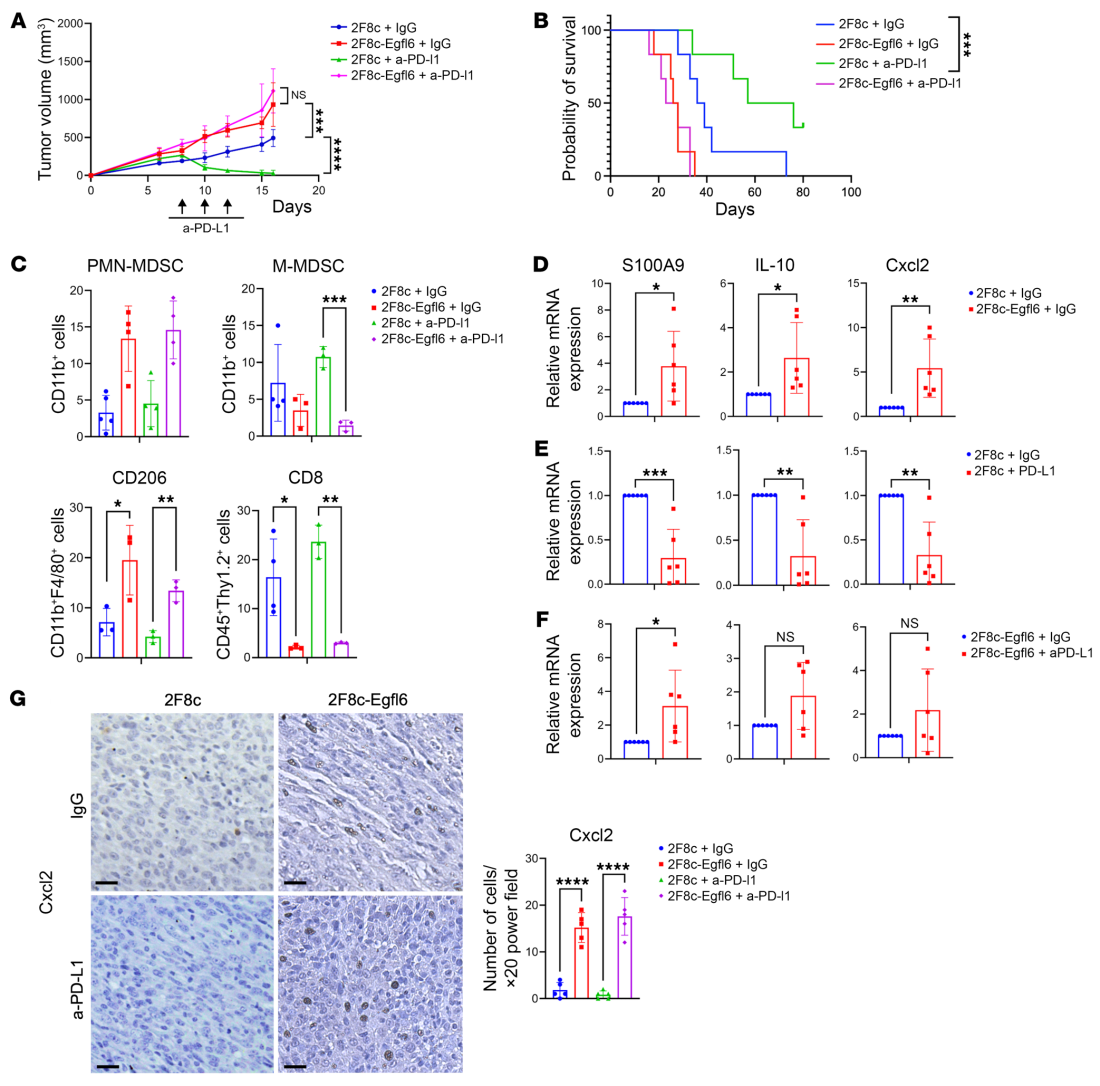
Tumor expression of Egfl6 induces resistance to anti-PD-L1 immunotherapy. (**A**) 2F8c and 2F8c-Egfl6 tumor growth in mice treated with anti-PD-L1 Ab or IgG isotype control Ab (*n* = 8 mice per group). *P < 0.05, 2F8c + IgG versus 2F8c-Egfl6 + IgG; ***P < 0.001, 2F8c + anti-PD-L1 versus 2F8c + IgG. (**B**) Kaplan-Meier survival analysis for the indicated treatment groups. ***P < 0.001, 2F8c + anti-PD-L1 versus 2F8c + IgG. Survival statistics were calculated using log-rank (Mantel-Cox) analysis from Kaplan-Meier survival plots. (**C**) Flow cytometry quantification of intratumoral PMN-MDSCs (CD11b^+^Ly6G^+^Ly6C^–^), M-MDSCs (CD11b^+^Ly6G^–^Ly6C^+^), CD206^+^ TAMs, and CD8^+^ T cells in the indicated tumors. (**D**–**F**) qPCR analysis of mRNA expression of *S100A9*, *IL-10*, and *Cxcl2* gene expression in (**D**) 2F8c-Egfl6 versus 2F8c, (**E**) anti-PD-L1–treated 2F8c versus IgG-treated 2F8c, (**F**) anti-PD-L1–treated 2F8c-Egfl6 versus IgG-treated 2F8c-Egfl6 tumor samples. (**G**) Representative images of IHC staining showing Cxcl2-expressing cells in control and a-PD-L1–treated tumor tissue sections. Graph represents the number of Cxcl2^+^ cells in the indicated tumors. Scale bars: 20 μm. Error bars show SEM. Experiments were performed in triplicate. Statistical significance was determined by unpaired 2-tailed *t* test, 1-way, or 2-way ANOVA with Tukey’s multiple comparisons test. *P < 0.05, **P < 0.01, ***P < 0.001, ****P < 0.001.

IPA analysis of our gene expression data indicated upregulation of PD-1/PD-L1 signaling in myeloid cells infiltrating Egfl6+ tumors (Figure 3B). Moreover, our data showed that Egfl6 enhanced the expression of PD-L1 in macrophages (Figure 3, C and D) and reduced the cytotoxic activity of CD8^+^ T cells (Figure 2H). Thus, we tested whether Egfl6 expression could impact the tumor response to a-PD-L1 immune therapy. We have previously demonstrated that 2F8c is an ICI-responsive tumor model (34, 35). 2F8c-Egfl6 and 2F8c cells were injected in WT mice. Tumors were allowed to engraft for 7 days, and then mice received i.p. injections of a-PD-L1 or isotype IgG. As expected, a-PD-L1 treatment significantly reduced tumor volume and improved the survival rate of 2F8c tumors compared to IgG-treated controls. Conversely, 2F8c-Egfl6 tumors demonstrated no response to immune therapy (Figure 4, A and B). Flow cytometry analysis indicated that the number of PMN-MDSCs and CD206^+^ TAMs were significantly higher in a-PD-L1-treated 2F8c-Egfl6 tumors compared to a-PD-L1-treated 2F8c tumors (Figure 4C). The number of M-MDSCs was lower in untreated and treated Egfl6+ tumors (Figure 4C). Consistent with an immunosuppressive phenotype of Egfl6, a-PD-L1-treated 2F8c-Egfl6 tumors showed a drastic reduction in the number of infiltrating CD8^+^ T cells compared to a-PD-L1-treated 2F8c tumors (Figure 4C).

Since *IL-10* and *Cxcl2* were upregulated in CD11b^+^ cells from Egfl6+ tumors (Figure 3A) and mediated Egfl6+ PMN-MDSC immunosuppressive functions in vitro (Figure 3F), we analyzed their expression in a-PD-L1-treated tumors. Consistent with the results described above, *S100A9, IL-10*, and *Cxcl2* were upregulated in Egfl6+ tumors (Figure 4D). While *S100A9, IL-10*, and *Cxcl2* expression was reduced in a-PD-L1-treated 2F8c tumors compared to tumor controls (Figure 4E), no reduction was detected between a-PD-L1-treated and control Egfl6+ tumors (Figure 4F). In line with the mRNA expression, IHC showed that Cxcl2 was abundantly expressed in non-tumor cells of a-PD-L1- and IgG-treated Egfl6-2F8c tumors compared to their control 2F8c tumors (Figure 4G).

### Egfl6 NAb restores response to a-PD-L1 therapy.

**Figure 5:**
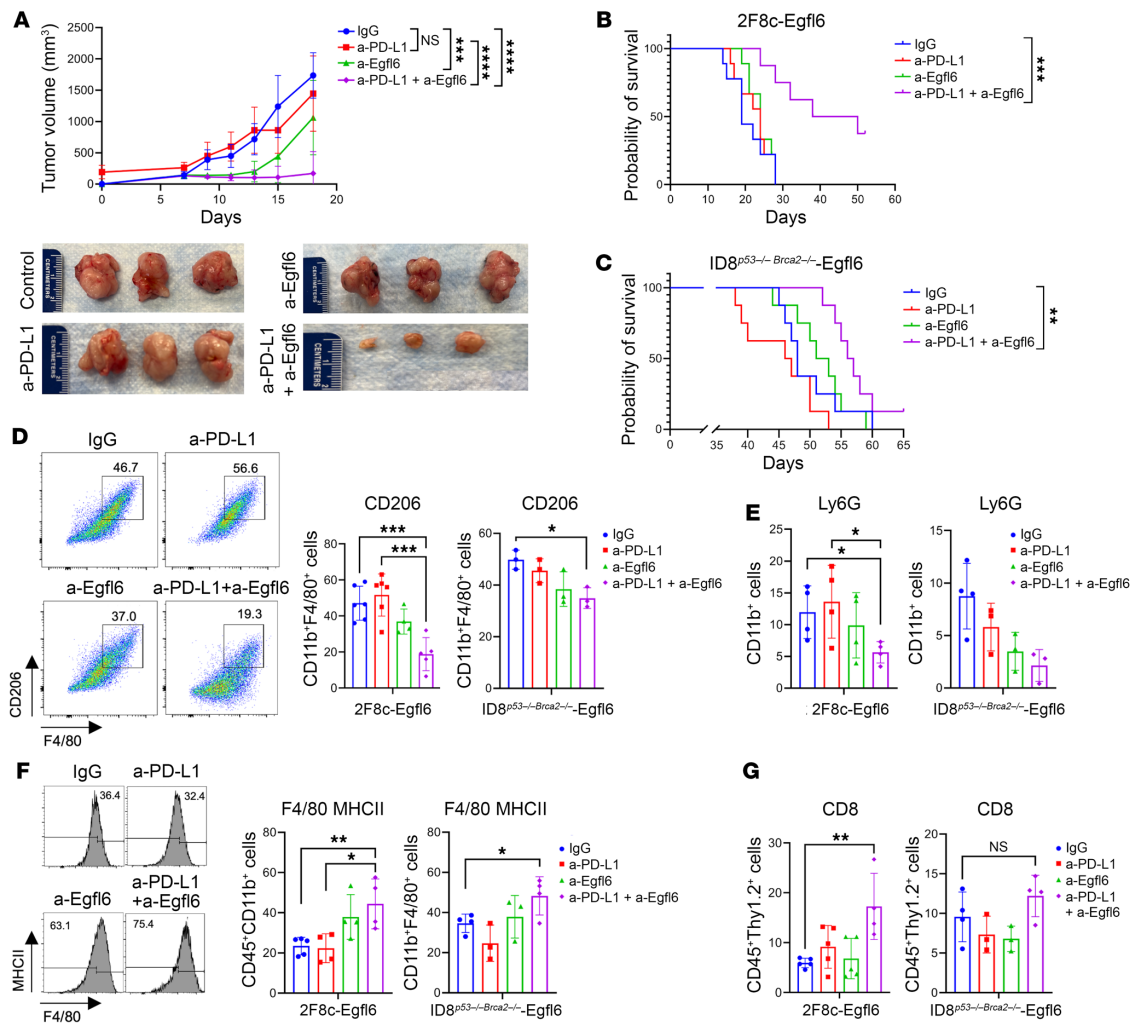
Combined treatment of a-Egfl6 and anti-PD-L1-induced high antitumor immune response. (**A**) Volume changes (mm^3^) and representative images of 2F8c-Egfl6 subcutaneous tumors treated with IgG isotype Ab (Control), a-PD-L1 Ab, and a-Egfl6 Ab, alone or in combination, were resected and measured 2 days after the last treatment (*n* = 8 mice per group). **P < 0.01, IgG Ab versus a-Egfl6 Ab; ***P < 0.001, anti-PD-L1 Ab versus a-Egfl6 Ab and IgG Ab versus anti-PD-L1+ a-Egfl6 Abs. (**B** and **C**) Kaplan-Meier overall survival analysis for 2F8c-Egfl6 (**B**) and ID8*^p53–/– Brca2–/—^*-Egfl6 (**C**) mice receiving the indicated treatment. Survival statistics were calculated using the Log-rank (Mantel-Cox) test analysis. (**D**–**G**) Flow cytometric gating and quantification of CD206^+^ TAMs (**D**), PMN-MDSC (CD11b^+^Ly6G^+^Ly6C^–^) (**E**), MHCII^+^ TAMs (**F**), and CD8^+^ T (CD45^+^Thy1.2^+^) (**G**) cells in 2F8c-Egfl6 and ID8*^p53–/– Brca2–/—^*-Egfl6 tumors. Error bars show SEM. Experiments were performed in triplicate. Statistical significance was determined by unpaired 2-tailed *t* test or 2-way ANOVA with Tukey’s multiple comparisons test. *P < 0.05, **P < 0.01, ***P < 0.001, ****P < 0.001.

Given that Egfl6 enhanced the expression of PD-L1 on tumor-infiltrating myeloid cells (Figure 3C) and inhibited the response to ICI therapy (Figure 4, A and B), we reasoned that targeting tumor-derived Egfl6 might reverse the immunosuppressive TME and restore the efficacy of a-PD-L1 treatment in a 2F8c tumor model. To test this hypothesis, we treated 2F8c-Egfl6 tumor-bearing mice with IgG control, a-PD-L1, and Egfl6 NAbs or a combination of Egfl6-NAb and a-PD-L1. Treatments with Egfl6 NAbs alone modestly reduced tumor growth (Figure 5A) but did not affect the probability of survival (Figure 5B). As above, a-PD-L1 had no effect on tumor growth. However, administration of a-Egfl6 combined with a-PD-L1 dramatically reduced tumor growth (Figure 5A) and prolonged the long-term survival of the mice (Figure 5B).

Next, we tested the efficacy of a-Egfl6 and a-PD-L1 alone or in combination in the murine OvCa ID8 cells with double deletion of TP53 and BRCA2 (ID8*^p53–/– Brca2–/–^*). Using lentiviral particles, we over-expressed *Egfl6* in these cells generating ID8*^p53–/– Brca2–/–^*-Egfl6. As control, we transduced cells with lentiviral particle control vector, generating ID8*^p53–/– Brca2–/–^*-CV. The expression of Egfl6 was confirmed via qPCR (Figure S5A). ID8*^p53–/– Brca2–/–^*-Egfl6 tumor-bearing mice treated with a-Egfl6 + a-PD-L1 showed a significantly prolonged survival (median survival = 57) compared with IgG isotype control (median survival = 48) (*P* value 0.0011). There was no significant difference between the group of mice receiving single treatment of a-Egfl6 (median survival = 52, *P* value 0.6967) or a-PD-L1 (median survival = 46.5, *P* value 0.1977) versus IgG isotype control (Figure 5C).

Analyses of tumor-infiltrating immune cells indicated that tumor-bearing mice receiving coadministration of a-Egfl6 and a-PD-L1 Abs displayed (a) a significant reduction of CD206^+^ TAMs (Figure 5D) and PMN-MDSCs (Figure 5E) and (b) an increased number of MHCII^+^ TAMs (Figure 5F) compared with mice receiving single treatment of a-Egfl6 Ab, a-PD-L1 Ab, or IgG isotype Ab control. In addition, a-Egfl6 + a-PD-L1 combination therapy increased the number of CD8^+^ T cells in the 2F8c-Egfl6 tumor model (Figure 5G, left panel), whereas no difference in total number of CD8^+^ T cells or specific subpopulations, such as CD8^+^ Teff (CD44^+^CD62L^–^) was detected in the ID8*^p53–/– Brca2–/–^*-Egfl6 model (Figure 5G, right panel and Supplemental Figure 5B).

Next, we evaluated whether CD8^+^ T cell depletion could inhibit the synergism of a-Egfl6 and a-PD-L1. We depleted CD8^+^ T cells in ID8*^p53–/– Brca2–/–^*-Egfl6 tumor-bearing mice receiving a-Egfl6 combined with a-PD-L1. No significant difference in survival was detected between the a-CD8 + a-Egfl6 + a-PD-L1- compared with a-Egfl6 + a-PD-L1-treated mice (Supplemental Figure 5C). This is consistent with our in vitro experiments showing that rEgfl6 did not directly modulate CD8^+^ T cell activities (Figure 1G).

We also tested whether double treatment with a-Egfl6 and a-PD-L1 Abs could affect the number of immune cells at other tissue sites. No significant difference was found in the BM between the double-treated and control groups (Supplemental Figure 5, D and E), indicating that the treatments mainly affected the number and phenotype of tumor-infiltrating immune cells.

**Figure 6:**
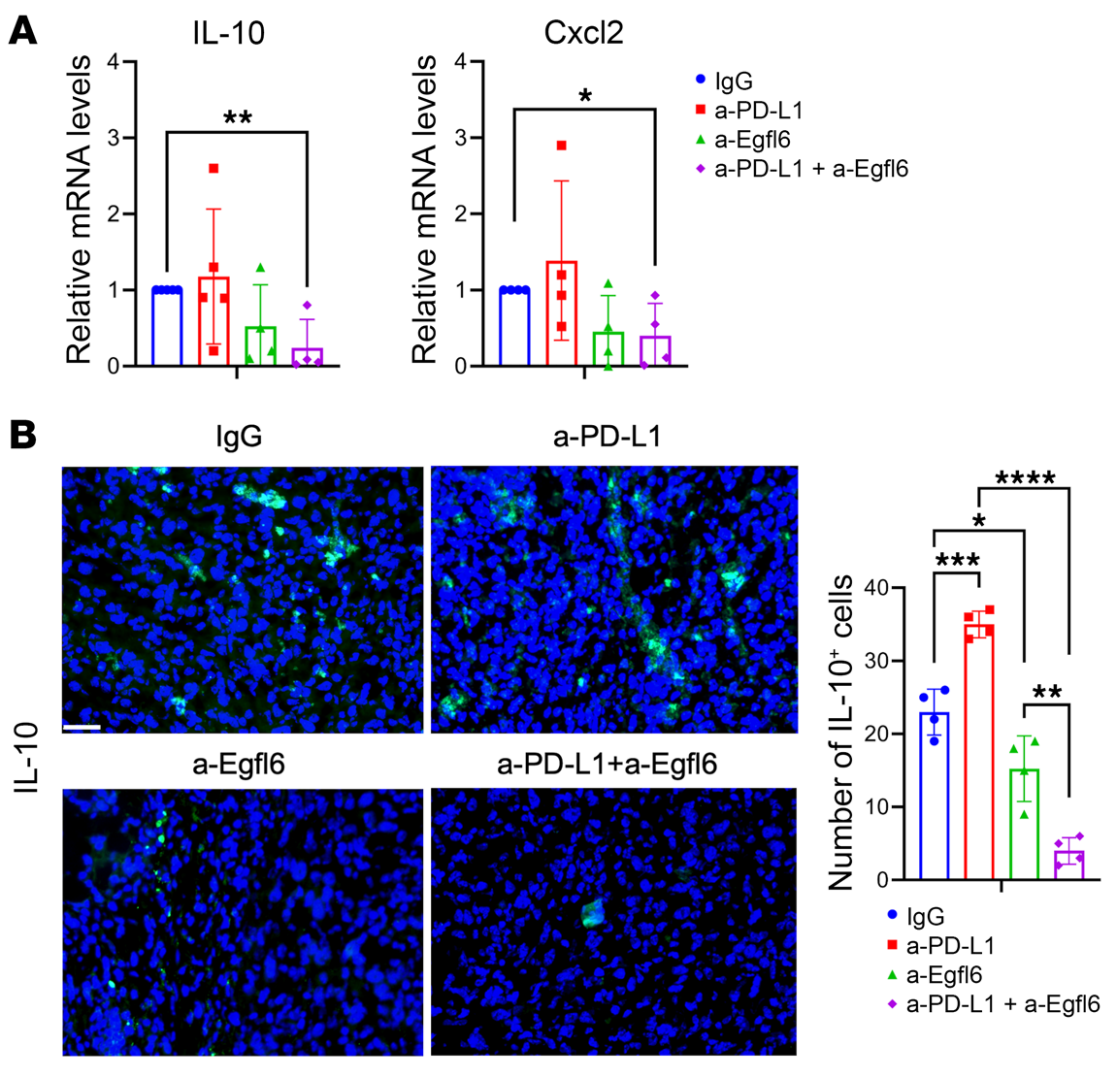
Combined treatment of a-Egfl6- and anti-PD-L1 reduced IL-10 and Cxcl2 expression. (**A**) qPCR analyses of *IL-10* and *Cxcl2* in the indicated treated Egfl6^+^ 2F8c tumors. (**B**) IF images and quantification of IL-10 expression in the indicated treated Egfl6^+^ 2F8c tumors. *P* values were calculated using unpaired 2-tailed *t* test. Data are presented as mean ± SEM. *P < 0.05, **P < 0.01, and ***P < 0.001 , ****P < 0.0001. All results are representative of 3 independent experiments. Scale bar: 30 μm.

Importantly, qRT-PCR and immunofluorescence analyses also revealed that double treatment, with both a-Egfl6 and a-PD-L1, reduced tumor expression levels of both IL-10 and Cxcl2 (Figure 6, A and B).

Altogether, our data suggest that Egfl6 signaling inhibition synergizes with PD-1/PD-L1 immune checkpoint blockade via modulation of myeloid cells, improving antitumor activity and survival in mice.

### EGFL6 induces an immunosuppressive phenotype of human MDSCs and TAMs via β3 integrin.

**Figure 7:**
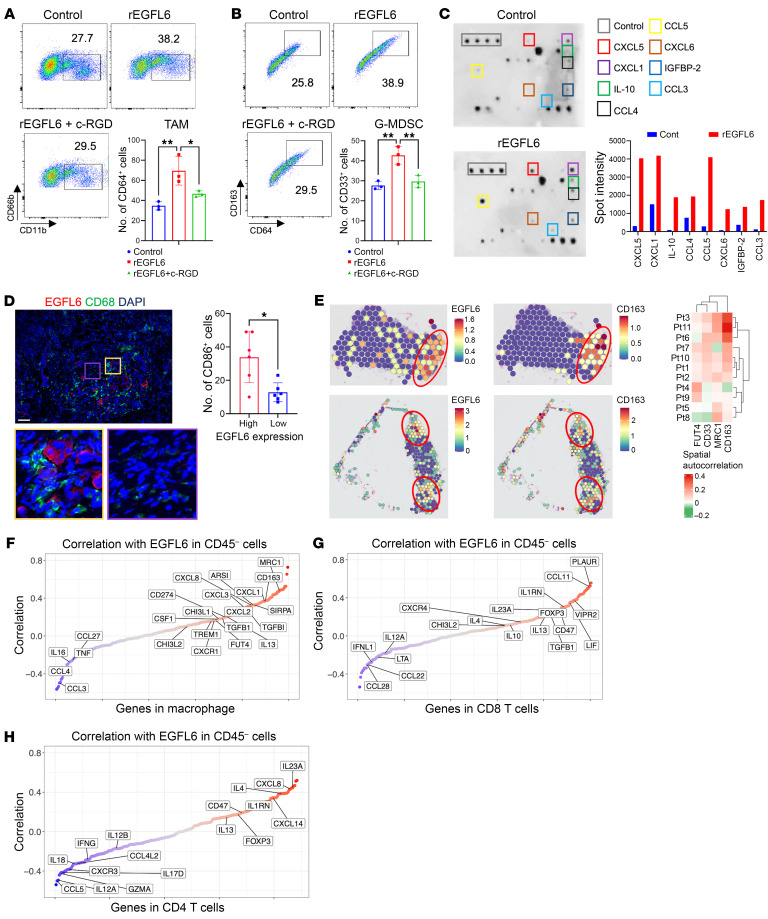
EGFL6 induces an immunosuppressive phenotype of human myeloid cells. (**A** and **B**) Gating and quantification of human CD11b^+^CD66b^+^ (**A**) and CD11b^+^CD163^+^CD64^+^ (**B**) cells in CD33^+^ cells isolated from ascites of patients with HGSOC and stimulated with rEGFL6 +/– c-RGD. (**C**) Cytokine array and densitometry of the CM of CD33^+^ ascites from patients with HGSOC stimulated with GM-CSF +/– rEGFL6. Spot intensities were calculated using ImageJ software. (**D**) Representative immunofluorescence images showing EGFL6 expression (red) and CD68 cell (green) localization in HGSOC tumor tissue sections (*n* = 6 per group). DAPI stained nuclei. Graph represents the number of CD68-positive cells in tissues expressing high or low levels of EGFL6. Scale bar: 100 μm. (**E**) Spatial feature plots of *EGFL6* and *CD163* markers and spatial autocorrelation of selected genes. Moran’s I test, implemented in the Seurat FindSpatiallyVariableFeatures function, was applied to compute spatial autocorrelation of the expression of each gene. Data are from a previously published dataset (55). (**F**–**H**) Sorted correlation plots between mRNA expression of EGFL6 in CD45^–^ cells and mRNA expression of cytokines and surface proteins in the indicated immune cells. Correlation was computed using the Spearman’s correlation with the sample-wise averaged gene expression. Each dot represents the Spearman’s correlation coefficients of a gene, and the dots were sorted in ascending order. *P* values were calculated using unpaired 2-tailed *t* test, 1-way, or 2-way ANOVA with Tukey’s post test for multiple comparisons. Data are presented as mean ± SEM. *P < 0.05, **P < 0.01, ***P < 0.001 and ****P < 0.0001.

Next, we evaluated the role of EGFL6 in the differentiation of human tumoral granulocytes and monocytes. As CD33 is highly expressed in myeloid cell progenitors (53, 54), we isolated CD33^+^ cells from human OvCa-associated ascites and treated them with human rEGFL6 protein for 48 hours. rEGFL6 increased the number of CD66b^+^ granulocytes (Figure 7A) and maturation of macrophages toward an immunosuppressive phenotype, defined as CD64^+^CD163^+^ (Figure 7B). As was shown previously, the addition of c-RGD compound inhibited the activity of EGFL6 on human myeloid cell differentiation (Figure 7, A and B).

To investigate whether EGFL6 could also affect myeloid cell cytokine and chemokine secretion, we performed a cytokine array of human CD33^+^ cells isolated from ascites from patients with OvCa and treated with rEGFL6 for 48 hours. EGFL6 enhanced the expression of factors associated with immunosuppression, including CXCL5, CXCL1, IL-10, CXCL6, and CCR5 ligands CCL3 and CCL4 (Figure 7C). Consistent with these results, coimmunofluorescence staining with EGFL6 and CD68 Abs in human HGSOC tissues showed that CD68^+^ cells were commonly adjacent to EGFL6-expressing cells (Figure 7D). To further validate these findings, we used OvCa spatial transcriptomics dataset from Stur et al. (55). Using the Moran’s I test, we found that the spatial location of EGFL6 spatially autocorrelated with *CD163^+^* and *Mrc1^+^* macrophages in 60%–70% samples and with CD33^+^ FUT4^+^ granulocytes in approximately 50% samples (Figure 7E).

Next, using integrative analysis single cell RNA sequencing (scRNA-seq) OvCa datasets (10, 56–58) we assessed whether EGFL6, expressed in CD45 negative cells, correlated with immune cell gene expression. Consistent with murine data and human spatial transcriptomic dataset, *EGFL6* mRNA expression was positively correlated with *CD163*, *MRC1*, *CXCL2*, *TREM1*, *CXCL8,* and *CD274* mRNA expression in macrophages (Figure 7F). In CD8^+^ and CD4^+^ T cells, *EGFL6* mRNA expression was positively correlated with mRNA expression of markers associated with immunosuppressive activities, including *IL4*, *IL13*, *IL1RN*, *IL23A*, and *FOXP3* (Figure 7, G and H)and negatively correlated with mRNA expression of activation markers such as *GZMA*, *IL12A*, *IFNG*, and *CXCR3* (Figure 7H). Interestingly, *EGFL6* was positively correlated with *CD47* mRNA expression in T cells and *SIRPA* in macrophages (Figure 7, F–H). Interaction of *CD47* with *SIRPA* inhibits the phagocytic activity of macrophages, overcoming the expression of ‘eat me’ signals and help tumor cells to evade macrophage surveillance.

## Discussion

Our group and others have previously reported the ability of EGFL6 to promote ovarian tumorigenesis by increasing angiogenesis, stimulating cancer cell asymmetric division and inducing migration and cancer cell metastasis (26, 29, 32, 33, 42). EGFL6 has now been shown to promote the growth of numerous tumor types, including breast, head and neck, and colorectal cancer (26, 28, 30, 42). Consistent with a protumorigenic role for EGFL6, increased EGFL6 expression is also associated with poor prognosis in ovarian, breast, and colorectal cancers (26, 28, 42). To date, there is no information on the impact of EGFL6 on immune cells. In this study, we provide evidence that EGFL6, in parallel with its effects on tumor cells, both increases myeloid cell migration and drives the differentiation of macrophages and granulocytes toward an immunosuppressive phenotype.

Using ID8 and 2F8c tumor cells, 2 syngeneic models of OvCa, we demonstrated that tumor cell expression of Egfl6 induces tumor growth and inhibits antitumor immune response via accumulation of intratumoral PMN-MDSCs and TAMs. These Egfl6-exposed myeloid cells upregulate secretion of immunosuppressive factors such as PD-L1, IL-10, and Cxcl2. Indeed, depletion of monocytic and granulocytic cells drastically delayed the growth-promoting effects of Egfl6.

A high number of both MDSCs and TAMs correlates with poor survival in many cancer types, including OvCa (8, 13, 59, 60). Both cell types promote angiogenesis and metastasis through production of several factors, including VEGF, MMP9, S100A8/9, and CXC-chemokines (61, 62). MDSCs and TAMs are also known to inhibit antitumor immune response, reducing the efficacy of ICIs such as PD-1/PD-L1 and CTL-4 (63–67). Moreover, TAM or MDSC depletion studies in animal models have been shown to reduce tumor growth and progression (7).

The interaction between cancer cells and myeloid cells is very complex and not fully understood. Although in the last decade several TME factors have been identified as key regulators of tumor-associated myeloid cells, it remains unclear what the tumor-secreted factors that regulate monocytic and granulocytic cell differentiation into protumorigenic and immunosuppressive TAMs and PMN-MDSCs are. The studies presented here indicate that Egfl6 is a potentially novel tumor factor that accelerates migration of MDSCs and TAMs to tumor sites and regulates their functional activities. Interestingly, we found that, while the number of PMN-MDSCs was higher in Egfl6^+^ tumors, the number of M-MDSCs was lower in untreated and treated Egfl6^+^ tumors. Thus, we speculate that Egfl6 promotes the rapid differentiation of M-MDSCs into macrophages. Additional studies will be needed to confirm this.

Gene expression profiling of murine intratumoral CD11b^+^ cells from Egfl6 tumors and ex vivo experiments revealed that Egfl6 promotes Cxcl2 expression in both PMN-MDSCs and TAMs. Mechanistic studies indicate that Egfl6-induced Cxcl2 expression is mediated by activation of β3 integrin and induction of Syk phosphorylation. This finding is consistent with prior studies showing that MAPK family members are frequently associated with migration and cytokine production in myeloid cells (68). It is known that, upon binding to the CXCR2 receptor, CXCL2 recruits MDSCs to the tumor sites in a paracrine and autocrine manner, promoting tumor progression (68–70). Thus, Cxcl2 likely plays a role in Egfl6-mediated recruitment of MDSCs. Notably, in patients with OvCa, CXCL2 correlates with MDSC infiltration, angiogenesis, and short overall survival (15, 71).

Egfl6^+^ tumor-infiltrating PMN-MDSCs were found to express higher levels of IL-10 than control tumors. IL-10 is a well-known immunosuppressive cytokine that targets diverse cells, such as macrophages, which display high levels of IL-10R. By reducing macrophage surface expression of MHC-II and CD86 and limiting their antigen-presenting functions, IL-10 drives an inefficient T cell immune response (72–74). Notably, neutralization of IL-10 and Cxcl2 secreted by PMN-MDSCs isolated from Egfl6^+^ tumors resulted in increased production of IFN-γ in CD8^+^ T cells. This suggests that both IL-10 and Cxcl2 mediate, at least in part, Egfl6-dependent tumor immunosuppression.

Consistent with a substantial immunosuppressive role for EGFL6, in the immune hot/immune-responsive 2F8c mouse model, Egfl6 completely eliminated the response to a-PD-L1 immunotherapy. In 2F8c-Egfl6 tumors, both IL-10 and Cxcl2 expression levels remained very high, despite a-PD-L1 treatment. This aligns with other studies showing that increased release of IL-10 in OvCa is associated with a highly immunosuppressive environment driving tumor progression with ICI therapy (75). Further, melanoma and lung tumors escaping from aPD1 therapy have shown accumulation of PMN-MDSCs with high expression of CXCR2-ligands, including CXCL2 (2). Importantly, our in vivo studies indicate that while a-Egfl6 therapy improved response to a-PD-L1 therapy in both the 2F8c (s.c.) tumor model and the ID8*^p53–/– Brca2–/–^* (i.p.) model, it was more efficacious in the immune hot 2F8c tumor model. This likely relates to both the presence of antitumor T cells in the 2F8c model and the unique TME of the s.c. versus i.p. models. The ascites TME in the i.p. model is highly hypoxic with increased concentration of immunosuppressive chemokines and high numbers of regulatory T cells and immunosuppresive myeloid cells, making it particularly resistant to immunotherapy (36, 76–78). Despite this, we found a-Egfl6 therapy was still able to improve treatment response in this model.

The combination treatment, Egfl6 NAb and a-PD-L1, was associated with (a) a reduced number of intratumoral PMN-MDSCs and TAMs, (b) reduced PMN-MDSC and TAM secretion of IL-10 and Cxcl2, and (c) increased tumor infiltration of antitumor MHC-II^+^ macrophages. The combined treatment increased the number of cytotoxic CD8^+^ T cells in the 2F8c model whereas no significant changes were found in the ID8*^p53–/– Brca2–/–^* tumor model. Consistent with that and a recent study (77), depletion of CD8^+^ T cells did not affect the survival rate of a-Egfl6 + a-PD-L1–treated ID8*^p53–/– Brca2–/–^* tumor bearing mice. This further confirmed that Egfl6 regulates the ovarian immune TME through myeloid cells.

In summary, we believe that this work expanded our knowledge of the crosstalk between tumor cells and immune cells, suggesting a potentially novel Egfl6-dependent signaling axis that drives recruitment and differentiation of immunosuppressive myeloid cells in the tumor, resulting in resistance to a-PD-L1 immunotherapy. Thus, Egfl6 is a promising target for improving the efficacy of immunotherapy in patients with OvCa.

## Methods

### Sex as a biological variable.

Our study exclusively examined female mice and female human specimens because OvCa is only present in females.

### Cell culture.

The 2F8 resistant-to-cisplatin (2F8c) mouse OvCa cell line was obtained by exposing the 2F8 cells, in vitro, to increasing concentrations (up to 10 μM) of cisplatin as described previously (34, 35). 2F8c cells were maintained in culture with 1 μM cisplatin. The ID8 mouse cancer cell line was a kind gift from George Coukos (Lausanne University Hospital, Lausanne, Switzerland). The ID8*^p53–/– Brca2–/–^* cell line generated by CRISPR/Cas9-mediated knockout was provided by Iain McNeish (Imperial College London, London, United Kingdom). Human SKOV3 were purchased from ATCC. Murine and human cell lines were cultured in DMEM and RPMI media, respectively, supplemented with 10% heat-inactivated FBS, 100 U/mL penicillin, and 100 mg/mL streptomycin. 10 μg/mL of insulin-transferrin-sodium selenite supplement (Roche) was added to ID8 and ID8*^p53–/– Brca2–/–^* cell lines as described previously (79). Cells were cleared from *Mycoplasma* contamination.

### Egfl6 mouse model.

Egfl6 transgenic mice were generated by the University of Michigan Transgenic Animal Model Core. LOXP-STOP-LOXP-Egfl6 was introduced into intron 1 of mouse Gt (ROSA)26Sor locus via homologous recombination to generate Rosa^LSL–Egfl6^. Constitutive expression of Egfl6 in all tissues was achieved by crossing Rosa^LSL–Egfl6^ with B6.C-Tg (CMV-cre)1Cgn/J (Jackson laboratory).

### Isolation and differentiation of murine and human myeloid cells.

Murine BM cells were isolated by flushing femurs and tibias of C57BL/6J mice with complete DMEM. Debris was removed by passaging the suspension through a 70 μm nylon sterile strainer (Greiner Bio-1). After 2 washes with PBS, CD11b^+^ cells were isolated using magnetic beads (Miltenyi Biotec) and 2.5 × 10^6^ cells were seeded on 6-well plates (Corning Costar). Cells were supplemented with 50 ng/mL recombinant mouse granulocyte macrophage colony stimulating factor (GM-CSF) (R&D System) alone or in combination with 200 ng/mL recombinant murine Egfl6 (rEgfl6) (Sino Biological) and cultured for 4 days in a humidified incubator at 37°C and 5% CO_2_. Human MDSCs were isolated from ascites of high-grade serous cancer patients using CD33^+^ magnetic microbeads (Miltenyi) and stimulated with 50 ng/mL human GM-CSF (R&D Systems) and 200 ng/mL recombinant human EGFL6 (rEGFL6) (Sino Biological). In some experiments, the following reagents were added to the media: 20 nM Cyclo-RGDfK (c-RGD) (Selleckchem S7844), and 200 nM R406 (Selleckchem).

### In vitro differentiation of murine MDSCs or M1/M2 macrophages.

Murine BM CD11b^+^ cells isolated from healthy C57BL/6J mice were differentiated into MDSCs by stimulation with murine recombinant proteins GM-CSF and IL-6 for 5 days. In other experiments, BM CD11b^+^ cells were differentiated into M1 or M2 macrophages by stimulation with M-CSF or GM-CSF, respectively, for 4 days, and then adding LPS + IFN (M1) or IL-4 (M2) for 48 hours.

### Egfl6 Ab purification and production.

Hybridoma cells were maintained in RPMI with 5% FBS. Supernatant was collected after 7–10 days, cells/debris were removed by centrifugation, and supernatant was loaded on Protein G Agarose (Millipore), washed, and Ab was eluted with 50 mM glycine, pH 2.7, neutralized to pH 7.2–7.4 with 1 M Tris (pH 9.0). Eglf6 Ab was given intraperitoneally at 10 mg/kg twice a week. Dosing was selected based on prior studies, pharmacokinetic studies, and toxicity studies (performed by Jackson laboratories) (26, 80).

### Cell transfections and lentiviral transduction.

2F8c cells were transfected with Egfl6-pCMV6-Entry vector or control vector (OriGene), using Lipofectamine 3000 Reagent (Invitrogen), following the manufacture’s protocol. Egfl6-expressing 2F8c (2F8c -Egfl6) and control cells were then selected using 400 μg/mL G418. Egfl6 and control lentiviral particles were generated from HEK293T with packaging plasmids PSPAX2 and pMD2.G and pLenti-Egfl6 or control vector pLenti-C-Myc-DDK-P2A-Puro (OriGene). Egfl6-expressing ID8 (ID8-Egfl6) and control ID8 cells were generated by lentiviral transduction followed by selection with 1 μg/mL puromycin. A human EGFL6-expressing SKOV3 cell line (SKOV3-EGFL6) was generated as described (24).

### Quantitative qPCR.

Tumor tissues were homogenized with QIAshredder (Qiagen). Total RNA was isolated from cell and tissue lysates, using RNeasy mini kit (Qiagen) according to the manufacturer’s instructions. A total of 500–1000 ng of RNA was reverse transcribed using the Superscript III First Strand Kit (Invitrogen). A total of 3 μL of RT products was used to amplify *IL-10, Cxcl2, Egfl6, S100A9*, *S100A8*, and *Arg*. *β-actin* was used as an internal control. qRT-PCR was performed using a SYBR Green PCR kit (BioRad) and a CFX384 Real PCR system (BioRad). Gene expression was determined using the 2^−Δ^ ^Ct^ method. All experiments were repeated 3 times in triplicate.

### Gene expression profiling.

Total RNA of CD11b^+^ cells isolated from (a) *Egfl6* and C57BL/6J mouse BM cells (*n* = 3 per group) and (b) 2F8c-Egfl6 and 2F8c tumor tissues (*n* = 3 per group) was isolated as indicated above. The myeloid innate immune response was examined using the nCounter Mouse Myeloid Innate Immunity Panel v2 (NanoString Technologies). The protocol was carried out at the University of Pittsburgh NanoString facility, using 75 ng of total RNA from each sample following their commercial protocol. Data were analyzed using the NanoStringDiff R-package, following the procedure described in the package’s instructions. Normalization of mRNA content for heatmap and volcano visualization purposes was performed by using the NanoString Data Normalization function, which adjusts for positive control size factors, background noise, and housekeeping gene size factors. Differentially expressed genes were detected by using the glm.LRT function. *P* values were adjusted for multiple comparison using the procedure of Benjamini and Hochberg. A gene was considered significantly overexpressed if associated with a *P*_adj_ < 0.01 and a logFC > 1.

### Western blotting.

Total proteins were extracted using Pierce RIPA buffer (Thermo Fisher Scientific) with protease inhibitors (Complete Protease Inhibitor Cocktail, Roche) and phosphatase inhibitors (Phosphatase Inhibitor Cocktail II, Sigma- Aldrich). Proteins were separated by SDS-PAGE and transferred to PVDF membranes. Prestained Seeblue Plus2 protein ladder was used for reference (Thermo Fisher Scientific). Blots were incubated with the primary Abs overnight at 4°C: p38 (D13E1, Cell Signaling cat. 8690), phospho-p38 MAPK (Thr180/Tyr182) (Cell Signaling cat. 4511), src (Cell Signaling cat. 2108), phopsho-src (Tyr416) (Cell Signaling cat. 2101), Jun (60A8, Cell Signaling cat. 9165), Actin (Proteintech, cat. 66009), IL-10 (R&D, cat. AF519), Cxcl2 (R&D, cat. MAB452), Syk (Abcam, cat. ab255701), and phospho-Syk (Tyr525, Tyr526) (Thermo Fisher Scientific, cat. PA5-106111). Membranes were then incubated with m-IgGk BP-HRP (sc-516102) or mouse a-rabbit IgG-HRP (sc-2357) secondary Abs (Santa Cruz) and developed with chemiluminescence reagents (SuperSignal West Femto or Pico Substrate, Pierce).

### Cytokine Array.

The supernatants of CD33^+^ cells isolated from ascites from patients with HGSOC were cultured with 20 ng/mL GM-CSF +/– 200 ng/mL rEGFL6 for 48 hours and tested for cytokines and chemokines, using a cytokine array (RayBiotech, AAH-CYT-C5). The procedure was performed according to the manufacturer’s instructions. The membranes were developed and the dots were quantified using ImageJ.

### ELISA.

IL-10, Cxcl2, Granzyme B, IFN-γ, and Perforin concentrations in the supernatants of differentiated BM myeloid cells or CD8^+^ T cells were measured using a mouse ELISA kit (R&D Systems and Biomatik) following the manufacturer’s protocol. Egfl6 concentrations in the supernatants of murine OvCa cell lines were determined using an ELISA Kit purchased from Biomatik. All points were done in duplicate, and the experiments were repeated at least 3 times. Samples were read in a microplate reader (Infinite 200 Pro, Tekan).

### OvCa mouse models.

C57BL/6J female mice were purchased from JAX laboratory. Mice 8–10 weeks old were injected s.c. with 5 × 10^6^ 2F8c or 2F8c-Egfl6 or i.p. with 4 × 10^6^ ID8, ID8-Egfl6, ID8*^p53–/– Brca2–/–^*-Egfl6 cells. S.c. tumor size was measured twice a week with a Vernier caliper, and mice were euthanized if tumor volume exceeded 2,000 mm^3^ or became ulcerated. Volumes were calculated using the formula V = 1/2 (L × W^2^), where L is length (longest dimension) and W is width (shortest dimension). For i.p. tumors, mice were euthanized when they developed ascites and had a weight increase of over 35% of their original weight on the day of tumor-cell injections. Tumor growth and body weight-gain curves were graphed in Prism 7 (GraphPad).

### In vivo Ab treatments.

S.c. injected mice were randomly divided into groups (10 mice per group) and treated i.p. with 150 μg a-PD-L1 mAb (10F.9G2, Bio X Cell cat. BE0101), alone or in combination with 10 mg/kg a-Egfl6. Control mice received isotype rat IgG2b (LTF-2, Bio X Cell cat. BE0090). Anti-Egfl6 was given twice a week for 3 weeks, starting on Day 7 after tumor-cell inoculation. Anti-PD-L1 and isotype control were administered 3 times every 2 days, starting on Day 10 after tumor cell inoculation, when the tumor size was approximately 300–400 mm^3^. For tumor cells injected i.p., a-Egfl6 and a-PD-L1 treatments started on Day 14 and Day 21, respectively. In some experiments, CD8^+^ T cells were depleted using 300 μg/mL a-CD8 (53-6.7, Bio X Cell cat. BE0004-1) 1 week before starting a-Egfl6 treatments.

Monocytes and granulocytes were depleted using a-Ly6G/Ly6C Ab (RB6-8C5, Bio X Cell cat. BE0075) given i.p. Specifically, 24 hours after 2F8c+/–Egfl6 tumor cell injections or 2 weeks after ID8+/–Egfl6 tumor cell injections, mice (*n* = 10) received 1 a-Ly6G/Ly6C 300 μg loading dose, followed by 200 μg every other day for 18 days. As control, mice received isotype rat IgG Ab (*n* = 10).

### Murine tumor dissociation.

Fresh tumors were isolated, minced in a petri dish on ice, and then enzymatically dissociated into single-cell suspension according to the protocol of Tumor Dissociation Kit, mouse (Miltenyi Biotec),and followed by mechanical dissociation using gentleMACS Dissociator. Cell suspensions were filtered through a 70-μm cell strainer. ACK lysing buffer (Gibco) was used for the lysis of red blood cells. Cell suspension was refiltered through a cell strainer and washed with FACS buffer (PBS containing 1% BSA and 0.5 EDTA mM) followed by staining for flow cytometry analysis.

### Flow cytometry analysis.

Murine tumors dissociated into single cells or BM-derived cells were washed with FACS buffer and stained with fluorescently labeled Abs and incubated at 4°C for 40 minutes. The following primary Abs were purchased from BioLegend: CD45 (30-F11), CD11b (M1/70), CD206 (MMR), MHCII (M5/114.15.2), Ly6G (1A8), Ly6C (HK1.4), PD-L1 (10F.9G2), CD3 (17A2), Thy1.2 (53-2.1 and 30-H12), CD8 (53-6.7), and PD1 (29F.1A12)—whereas F4/80 (BM8) and CD11c (N418) were purchased from eBioscience. Human myeloid cells were stained with the following Abs from BioLegend: CD14 (63D3), CD64 (10.1), CD163 (RM3/1), CD33 (WM53), and CD66b (6/40C); and from eBioscience: HLA-DR (LN3). Multicolor FACS analysis was performed on a BD LSRII analyzer. All data analysis was performed using the flow cytometry analysis program FlowJo (Tree Star).

### ChIP Assay.

The ChIP assay was performed on fresh CD11b^+^ cells isolated from ID8+/–Egfl6 ascites, utilizing the commercially available ChromaFlash High Sensitivity ChIP Kit (Epigentek), according to the manufacturer’s instructions. Cells were fixed with 1% formaldehyde for 10 minutes at 37°C. Chromatin was precipitated with 4 μg of a-Jun (Cell Signaling) at 4°C overnight. The presence of *IL-10* gene promoter sequences in immunoprecipitated DNA was identified by RT-PCR using the following primer sequences: Fwd: TGTGCTTGCTGCTGGTAGAA, Rev: GCTACACGTCCTGTTGACCA. In control samples, primary Ab was replaced with nonimmune IgG. All experiments were repeated at least 3 times.

### Migration assay.

Migration studies were conducted using 24-well transwells, 5 mm pore size (Costar Transwell, Corning). In each well, 5 × 10^4^ stimulated murine or human myeloid cells were plated into the upper chamber in serum-free medium, whereas 700 μL of 5% FBS-containing medium was added in the lower chamber. In specific experiments, 6 × 10^5^ control or Egfl6-expressing cancer cells were plated in the lower chamber. After 16 hours, transwells were washed with PBS, and cells remaining in the upper chamber were removed using cotton swabs. Cells adhering to the lower surface were fixed with 4% paraformaldehyde for 20 minutes and stained with crystal violet 1%. Cells were then counted in 3 different fields, using a microscope at 20 × magnification.

### Patients and tissue sample immunofluorescence.

Six biopsies of patients with HGSOC were selected and collected at the Department of Obstetrics, Gynecology and Reproductive Science, University of Pittsburgh. The study was approved by the Institutional Review Board (IRB) of the University. Fresh tissues were embedded in OTC (optimal cutting temperature) compounds and stored at –80°C. Tissue sections of 4 micrometers were then fixed in 4% paraformaldehyde (20 minutes), permeabilized in 0.5% Triton-X100 (20 minutes) and blocked in 2% BSA (60 minutes). Tissues were then incubated with primary Abs at 4°C overnight followed by secondary a-mouse Alexa-488 or Cy3 Ab (Invitrogen Life Technologies) for 1 hour at room temperature. Abs were diluted in 1% BSA. Nuclei were stained with mounting medium containing DAPI (Vector Laboratories). Confocal images were captured on a Leica DM4 microscope. Images were acquired using a Leica DFC7000T camera and Leica Application Suite X.

### scRNA-Seq data analysis.

Four public scRNA-seq datasets were obtained from the Gene Expression Omnibus (GEO) database and the European Genomephenome Archive (EGA) database, under accession numbers GSE17368219 (56), GSE18488020 (57), GSE232314 (58), and EGAS0000100493514 (10). Selected samples were processed and integrated (*n* = 20), following the same workflow as in ref. 58. Cells were then assigned to one of the following cell types based on the expression of the marker genes: B cells (*CD79A*, *CD19*, *MS4A1*), CD4^+^ T cells (*CD3D* and *CD4*), CD8^+^ T cells (*CD3D* and *CD8A*), dendritic cells (*CD40*, *CD1C*, and *ITGAX*), macrophages (*CD14*, *CD68*, and *FCGR1A*), NK cells (*KLRB1*, *NCAM1*), and CD45^–^ cells (PTPRC^–^). For each cell population, sample-wise averaged gene expression was computed. The average expression of cytokines and surface proteins (81–83) in each CD45^+^ cell population was then correlated with the average *EGFL6* expression in the CD45^–^ cell population, using the Spearman’s Rho Statistics.

### Spatial transcriptomics data analysis.

The spatial transcriptomics dataset comprised 12 samples from 1 dataset (55), and 11 high-quality samples were selected for the subsequent analysis. Each raw count matrix was loaded as a Seurat object to include features detected in at least 3 spots and spots with at least 400 features. For each Seurat object, we applied SCTransform normalization followed by PCA. For each sample, we used the Seurat function FindSpatiallyVariableFeatures to identify features whose variability in expression was explained to some degree by spatial location, and we used the Moran’s I test (62) to compute the spatial autocorrelation of each gene by patient.

### Statistics.

Differences between 2 conditions were analyzed by 2-tailed *t* test, 1-way, or 2-way ANOVA. In all cases, *P* < 0.05 was considered statistically significant. Statistics were calculated using Prism software (GraphPad).

### Study approval.

Mice were maintained in accordance with institutional policies, and all studies were performed with approval of the Institutional Animal Care and Use Committee of the University of Pittsburgh.

### Data availability.

Values for all data points in graphs are reported in the Supporting Data Values file.

## Author contributions

RJB and SC conceived and designed the experiments. SHS, JAI, SB, NGJ, NM, and SC performed, at least in part, the in vitro, ex vivo, and in vivo experiments. CC acquired and analyzed Nanostring data. LZ and HUO acquired and analyzed scRNA-Seq and spatial transcriptomic datasets. EJ provided guidance and scientific discussions. SHS, SB, RJB, and SC assembled the data and wrote the original draft. All authors reviewed, edited, and approved the final version of the manuscript. The order of cofirst authors was determined by relative amount of data each contributed.

## Supplementary Material

Supplemental data

Unedited blot and gel images

Supporting data values

## Version 1 09/23/2024

In-Press Preview

## Version 2 11/01/2024

Electronic publication
