# Supplementary material for: Egfl6 promotes ovarian cancer progression by enhancing the immunosuppressive functions of tumor-associated myeloid cells
Source: J Clin Invest. 2024 Nov 1;134(21):e175147. doi: 10.1172/JCI175147 (PMC11527450; doi:10.1172/JCI175147)
Supplement: Supplemental data [file jci-134-175147-s202.pdf]

Supplemental Figures

Supplemental Figure S1

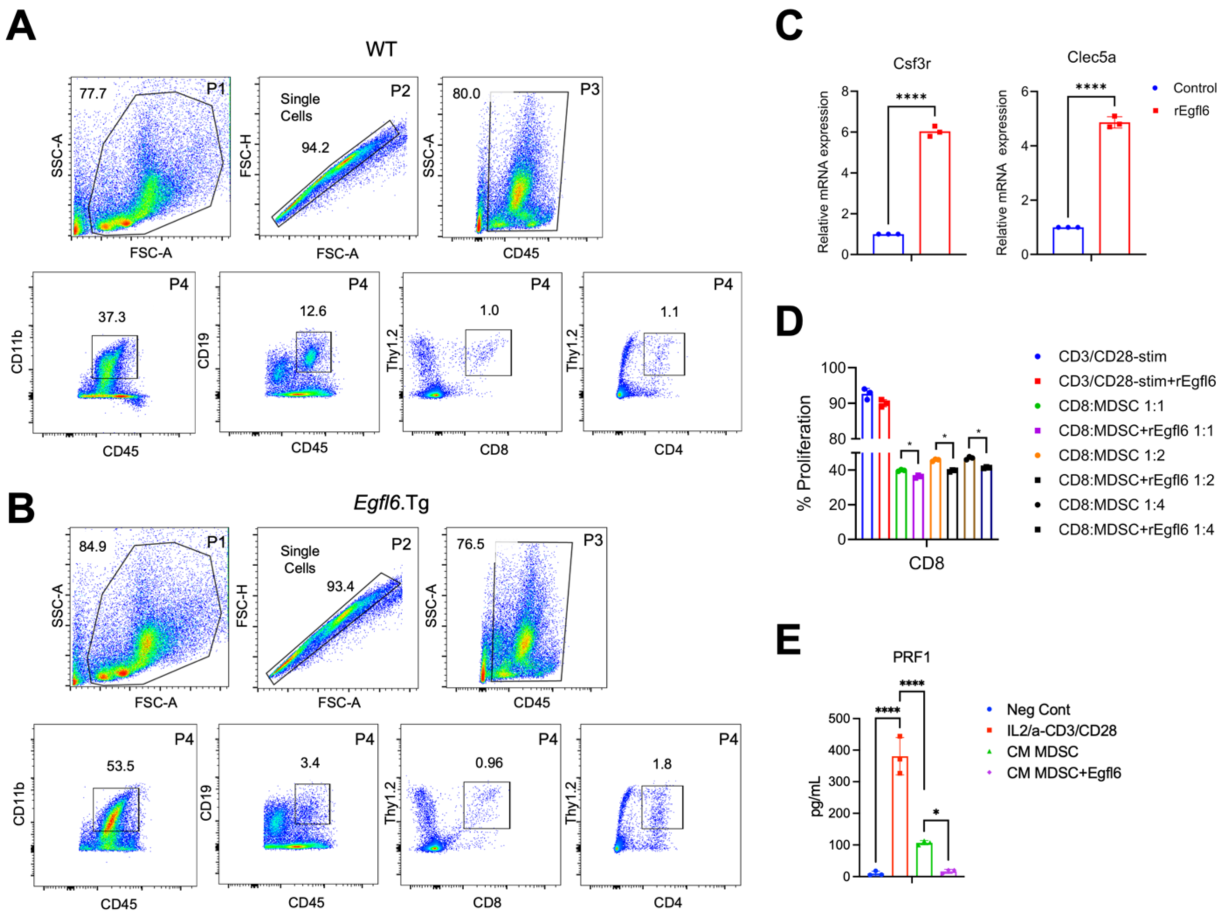

Supplemental Figure S1. Egfl6 modulates MDSC phenotype. A-B) Representative gating of CD11b^{+}, B, CD8, and CD4 T cells in BM of WT (A) and Egfl6 transgenic mice (Egfl6.Tg) mice (B). **C)** qPCR analyses of *Csf3r* and *Clec5a* in sorted BM CD11b^{+} cells stimulated with rGM-CSF + rEgfl6. Stimulation with rGM-CSF alone was used as control. **D)** Graph represents the % proliferation of IL-2 + CD3/CD28 activated CD8 T cells and cultured directly with rEgfl6-induced BM-derived MDSC or MDSC cells at different ratio. **E)** ELISA assay of Perforin 1 (PRF1) in IL-2 + CD3/CD28 activated CD8 T cells and cultured with the conditioned media (CM) of rEgfl6- stimulated BM-derived MDSC cells or MDSC control. Unstimulated CD8 T cells were used as negative control (Neg Cont). Experiments were completed in triplicate. *P* values were calculated using unpaired two-tailed *t* test or ordinary one-way ANOVA analyses. Data are presented as mean ± SEM. *$P < 0.05$, **$P < 0.01$, ***$P < 0.001$, ****$P < 0.0001$.

Supplemental Figure S2

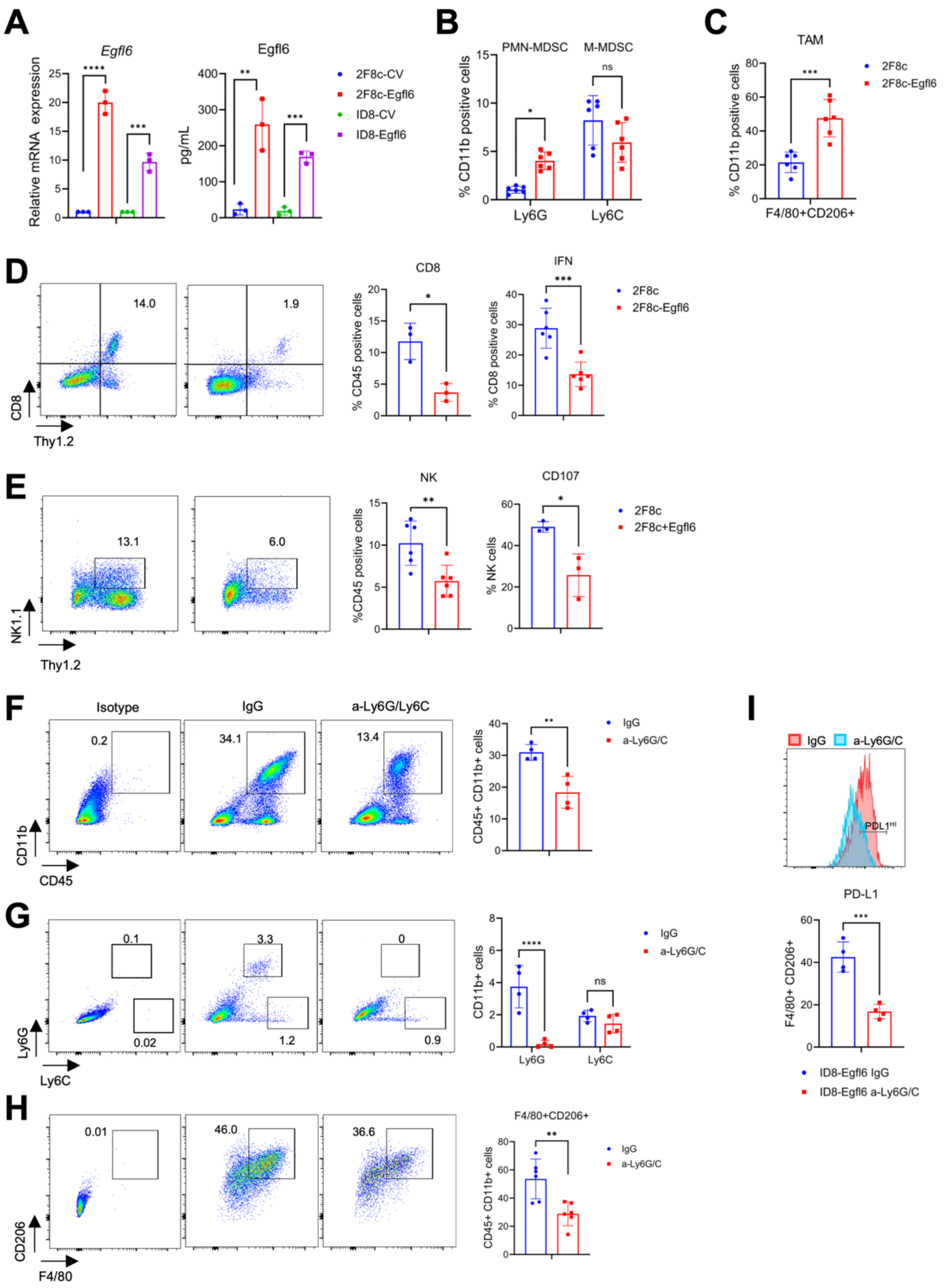

Supplemental Figure S2. Egfl6 induces accumulation of tumor-infiltrating myeloid cells and reduces the number of activated CD8 T and NK cells. A) Graphs showing qPCR analyses of *Egfl6* (left panel) and ELISA quantification of secreted Egfl6 protein (right panel) analyses in

2F8c+/-Egfl6 and ID8+/-Egfl6 cells. **B-C**) Graphs showing the quantification of (B) PMN-MDSCs ($CD11b^{+}Ly6G^{+}Ly6C^{-}$), M-MDSCs ($CD11b^{+}Ly6G^{-}Ly6C^{+}$), and (C) TAMs ($CD11b^{+}F4/80^{+}CD206^{+}$) in 2F8c+/-Egfl6 tumors (n=6 mice per group). **D-E**) Flow cytometric evaluation and quantification of (D) CD8 T ($CD45^{+}Thy1.2^{+}$) and (E) NK cells and their expression of IFN-g and CD107, respectively, in 2F8c+/-Egfl6 tumors (n=6 mice per group). **F-H**) Flow cytometric quantification of (F) myeloid cells ($CD45^{+}CD11b^{+}$), (G) PMN-MDSCs ($CD11b^{+}Ly6G^{+}Ly6C^{-}$), M-MDSCs ($CD11b^{+}Ly6G^{-}Ly6C^{+}$), and (H) $CD206^{+}$ TAMs ($F4/80^{+}CD206^{+}$) in ID8-Egfl6 ascites treated with anti-Ly6G/Ly6C or IgG isotype control. **I**) Gating and quantification of $CD206^{+}$ TAMs expressing PD-L1. Experiments were completed at least in triplicate. *P* values were calculated using unpaired two-tailed *t* test or two-way ANOVA analyses. Data are presented as mean ± SEM. *$P < 0.05$, **$P < 0.01$, ***$P < 0.001$ and ***$P < 0.0001$.

Supplemental Figure S3

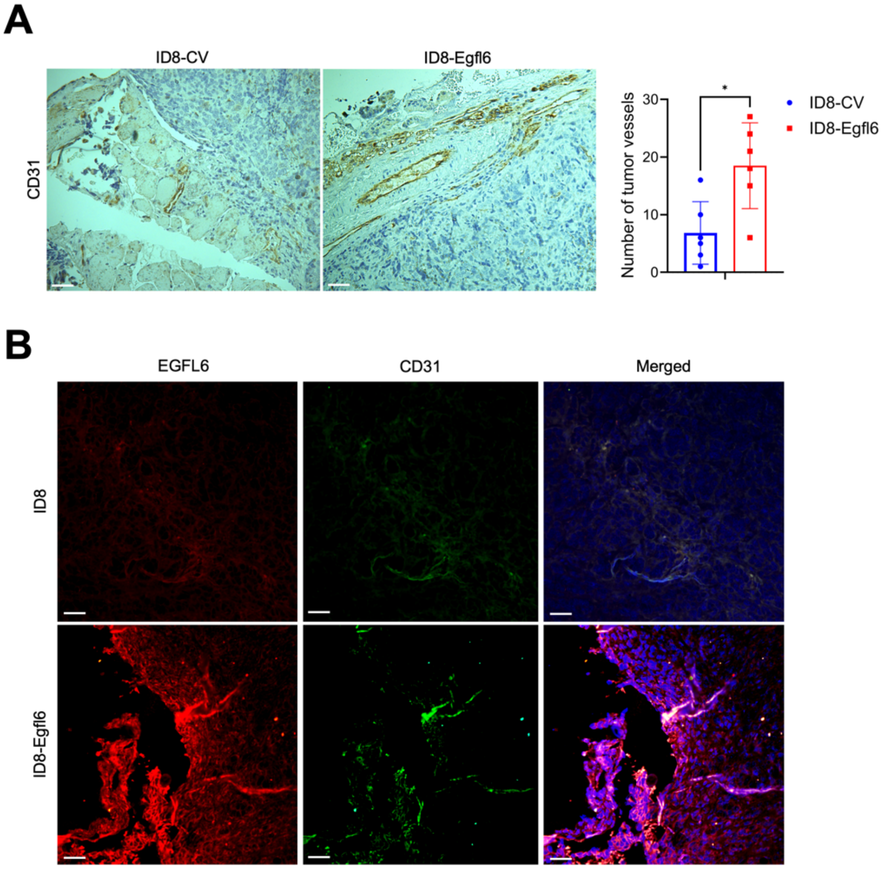

Supplemental Figure S3. Tumor Egfl6 increased the number of CD31$^+$ cells. A) Representative immunohistochemistry images and quantification of CD31 expression in ID8-CV and ID8-Egfl6 tissue samples (n=6 per group). *P* values were calculated using unpaired two-tailed *t* test. Data are presented as mean ± SEM. *$P < 0.05$. **B)** Representative immunofluorescence images showing the co-expression of Egfl6 in endothelial cells (CD31$^+$ cells) (n=3 per group). Scale bars 100µm.

Supplemental Figure S4

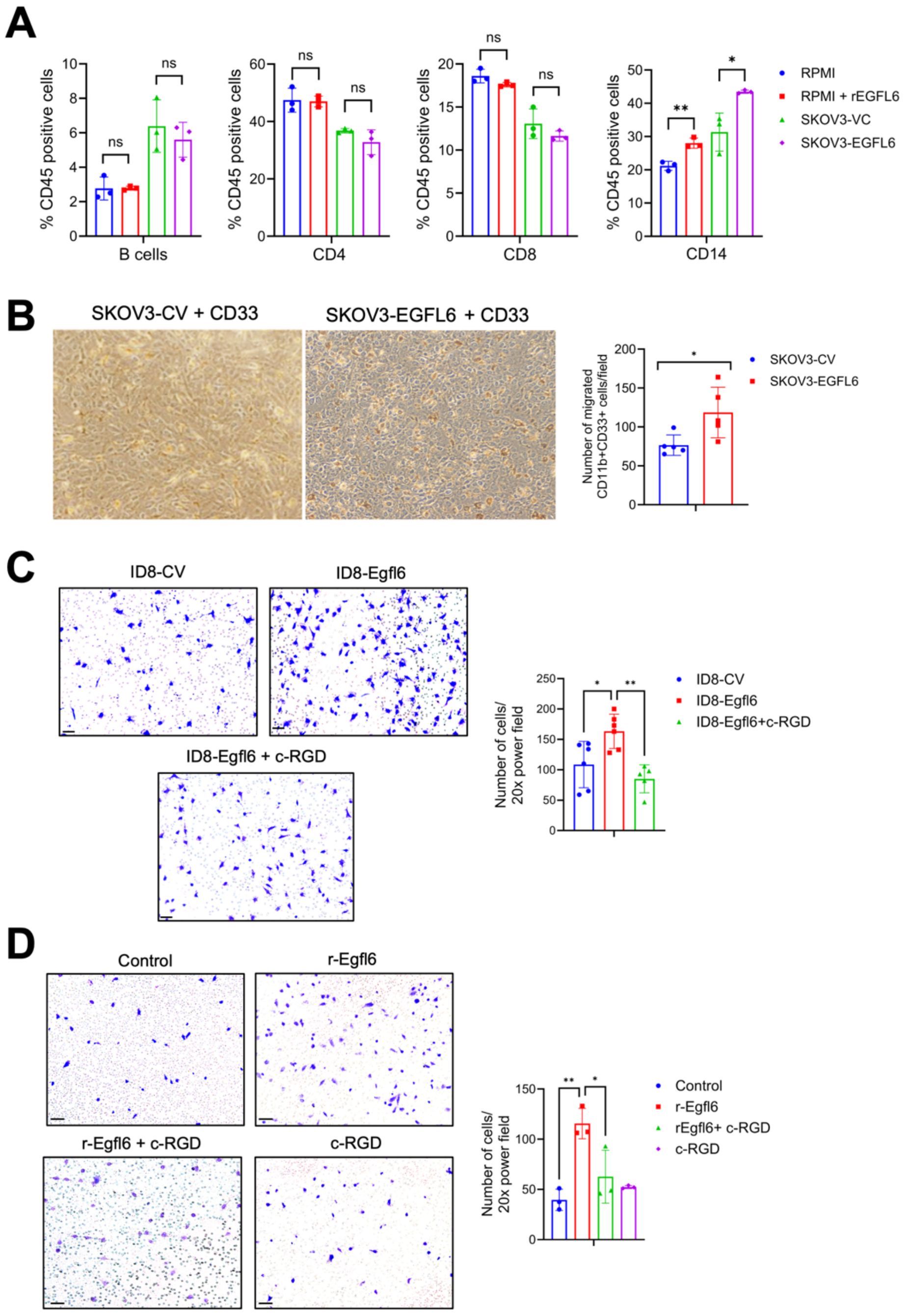

Supplemental Figure S4. Egfl6 induces migratory activities in myeloid cells via β3 integrin. A) Graphs represent flow cytometry quantification of Transwell-migration assay showing the number of PBMCs (top chamber) that migrated toward complete RPMI media +/- rEGFL6 (bottom chamber). **B)** Representative IHC images and quantification of anti-CD11b on HGSOC ascites $CD33^{+}$ migrated cells toward SKOV3-CV or SKOV3-EGFL6. **C-D)** Representative images and quantification of Transwell-migration assay showing the number of BM-isolated

$CD11b^+$ cells seeded onto the top chamber migrated toward: (C) ID8-CV or ID8-Egfl6 cells plated in the lower chamber; (D) or complete media containing rEgfl6. In indicated experiments, c-RGD, a β3 integrin inhibitor, was added to the bottom chamber. An average of five fields (20X) were acquired from each sample. Scale bars, 20 μm. Experiments were completed at least in triplicate. Data are presented as mean ± SEM, results were analyzed using unpaired two-tailed *t* test or ordinary one-way ANOVA. $^*P < 0.05$, $^{**}P < 0.01$.

Supplemental Figure S5

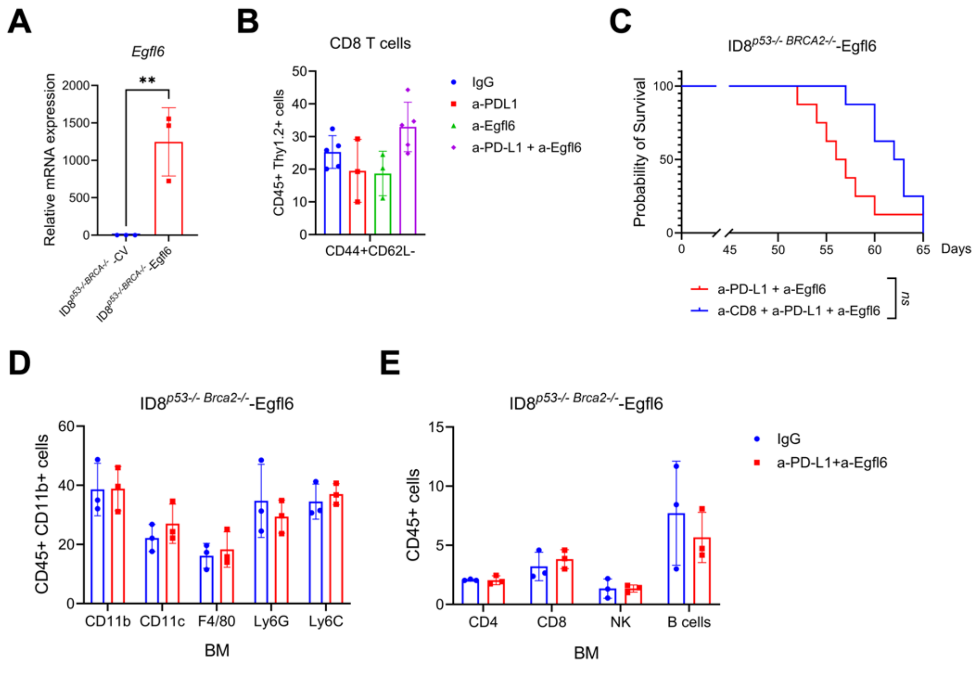

Supplemental Figure S5. CD8 T cell depletion does not affect a-Egfl6 + a-PD-L1 in ID8$^{p53-/-Brca2-/-}$-Egfl6 tumors. A) Graph shows qPCR analysis of *Egfl6* expression in ID8$^{p53-/-Brca2-/-}$-Egfl6 *vs* ID8$^{p53-/-Brca2-/-}$-CV cells. **B**) Flow cytometry quantification of CD44^{+}CD62L^{-} CD8 T cells in ID8$^{p53-/-Brca2-/-}$-Egfl6 tumors receiving the indicated treatments. **C**) Kaplan-Meier survival analysis for ID8$^{p53-/-Brca2-/-}$-Egfl6 tumor bearing mice receiving the dual treatment, a-Egfl6 + a-PD-L1, or the triple treatment, a-CD8 + a-Egfl6 + a-PD-L1. Survival statistics were calculated using log-rank (Mantel-Cox) analysis from Kaplan-Meier survival plots. **D-E**) Flow cytometry quantification of the indicated immune cells isolated from BM of ID8$^{p53-/-Brca2-/-}$-Egfl6 tumor bearing mice treated with a-Egfl6 + a-PD-L1Abs or IgG isotype control. Results were analyzed using unpaired two-tailed t-test or two-way ANOVA. Experiments were completed at least in triplicate. Data are presented as mean ± SEM. $^{**}P < 0.01$.
