# Supplementary figures and images for: Egfl6 promotes ovarian cancer progression by enhancing the immunosuppressive functions of tumor-associated myeloid cells
Source: J Clin Invest. 2024 Nov 1;134(21):e175147. doi: 10.1172/JCI175147 (PMC11527450; doi:10.1172/JCI175147)

Figure 3D

TAM

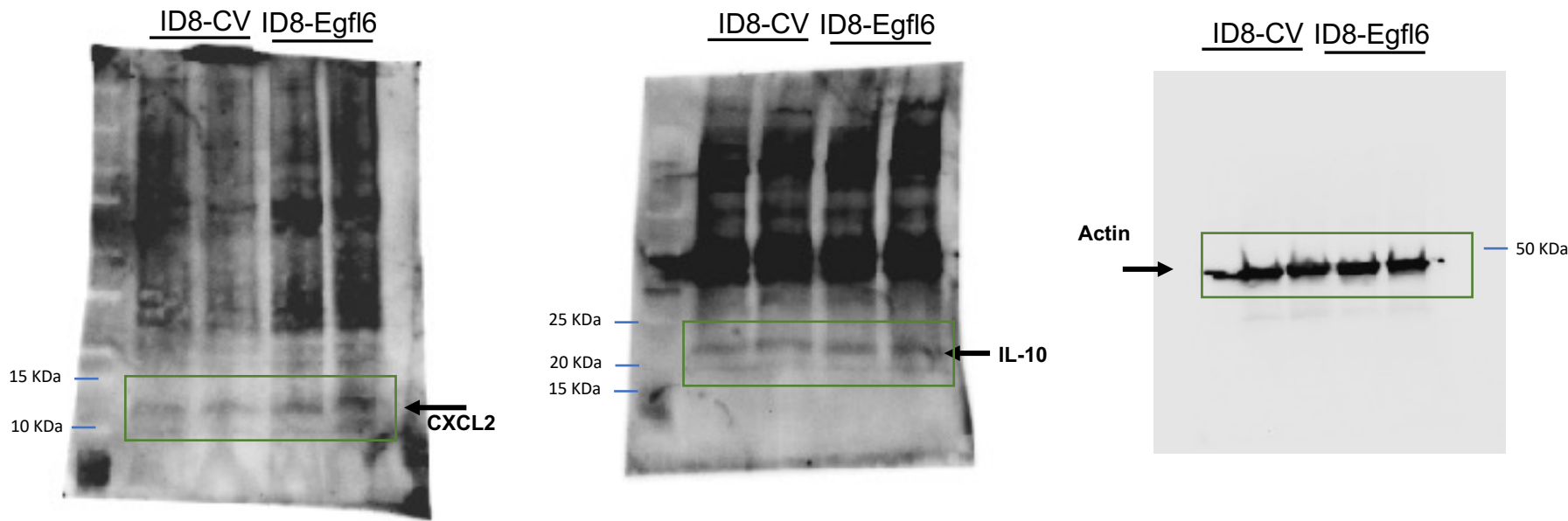

D

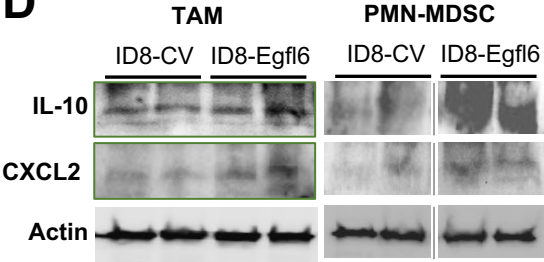

PMN-MDSC

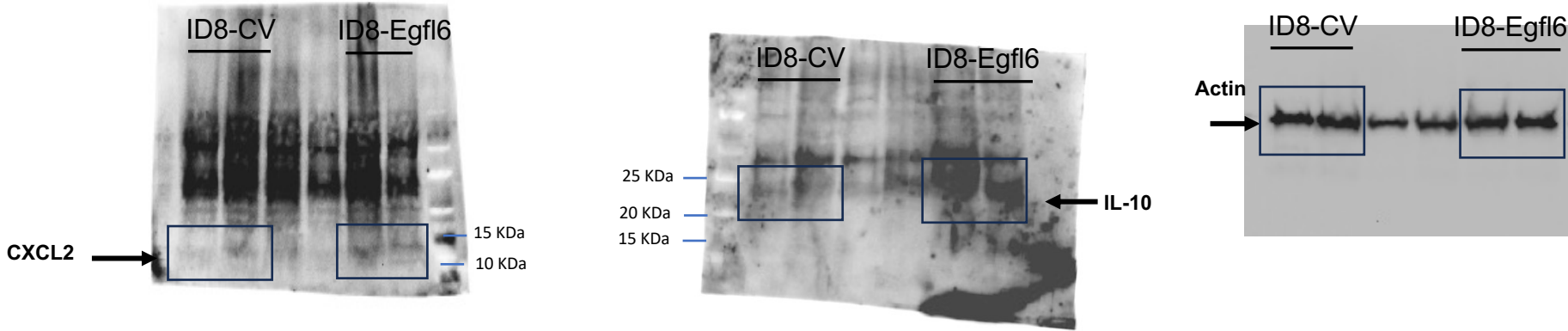

**FIGURE 3F**

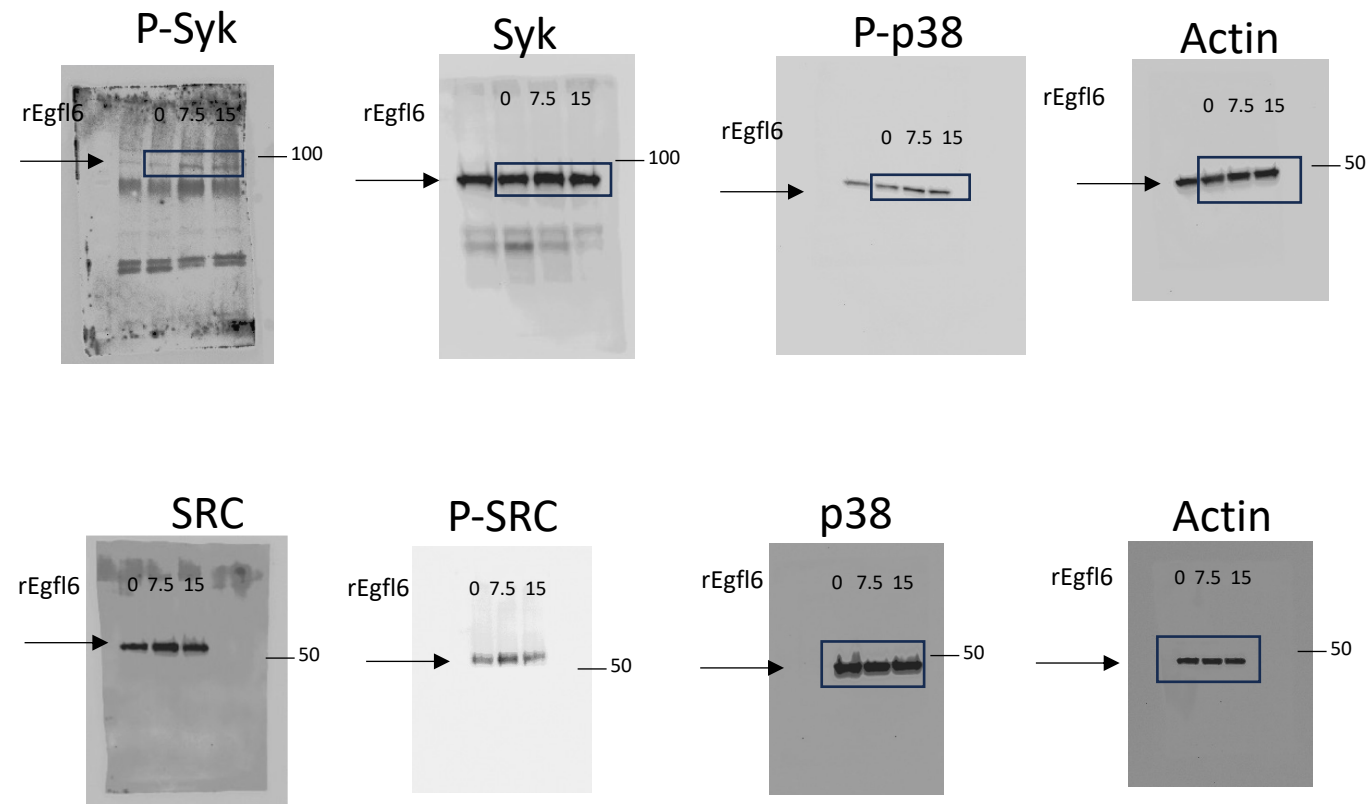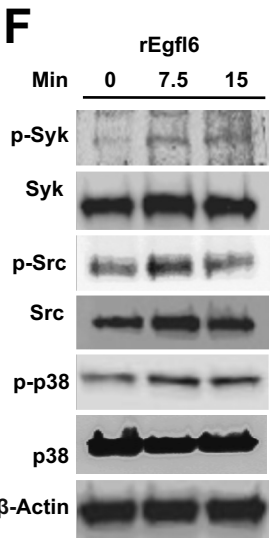

Supplement: Unedited blot and gel images [file jci-134-175147-s203.pdf]
